# Impaired AIF-CHCHD4 interaction and mitochondrial calcium overload contribute to auditory neuropathy spectrum disorder in patient-iPSC-derived neurons with *AIFM1* variant

**DOI:** 10.1038/s41419-023-05899-6

**Published:** 2023-06-26

**Authors:** Yue Qiu, Hongyang Wang, Mingjie Fan, Huaye Pan, Jing Guan, Yangwei Jiang, Zexiao Jia, Kaiwen Wu, Hui Zhou, Qianqian Zhuang, Zhaoying Lei, Xue Ding, Huajian Cai, Yufei Dong, Lei Yan, Aifu Lin, Yong Fu, Dong Zhang, Qingfeng Yan, Qiuju Wang

**Affiliations:** 1 College of Life Sciences, Zhejiang University, Hangzhou, Zhejiang 310058 China; 2 Senior Department of Otolaryngology, Head and Neck Surgery, Chinese PLA Institute of Otolaryngology, Chinese PLA General Hospital, Beijing, 100853 China; 3 Department of Pediatrics, The First Affiliated Hospital of Zhejiang University School of Medicine, Hangzhou, Zhejiang 310003 China; 4 The Children’s Hospital of Zhejiang University School of Medicine, Hangzhou, Zhejiang 310052 China; 5 Key Laboratory for Cell and Gene Engineering of Zhejiang Province, Hangzhou, Zhejiang 310058 China

**Keywords:** Apoptosis, Mechanisms of disease, Induced pluripotent stem cells

## Abstract

Auditory neuropathy spectrum disorder (ANSD) is a hearing impairment caused by dysfunction of inner hair cells, ribbon synapses, spiral ganglion neurons and/or the auditory nerve itself. Approximately 1/7000 newborns have abnormal auditory nerve function, accounting for 10%-14% of cases of permanent hearing loss in children. Although we previously identified the *AIFM1* c.1265 G > A variant to be associated with ANSD, the mechanism by which ANSD is associated with *AIFM1* is poorly understood. We generated induced pluripotent stem cells (iPSCs) from peripheral blood mononuclear cells (PBMCs) via nucleofection with episomal plasmids. The patient-specific iPSCs were edited via CRISPR/Cas9 technology to generate gene-corrected isogenic iPSCs. These iPSCs were further differentiated into neurons via neural stem cells (NSCs). The pathogenic mechanism was explored in these neurons. In patient cells (PBMCs, iPSCs, and neurons), the *AIFM1* c.1265 G > A variant caused a novel splicing variant (c.1267-1305del), resulting in AIF p.R422Q and p.423-435del proteins, which impaired AIF dimerization. Such impaired AIF dimerization then weakened the interaction between AIF and coiled-coil-helix-coiled-coil-helix domain-containing protein 4 (CHCHD4). On the one hand, the mitochondrial import of ETC complex subunits was inhibited, subsequently leading to an increased ADP/ATP ratio and elevated ROS levels. On the other hand, MICU1-MICU2 heterodimerization was impaired, leading to _m_Ca^2+^ overload. Calpain was activated by _m_Ca^2+^ and subsequently cleaved AIF for its translocation into the nucleus, ultimately resulting in caspase-independent apoptosis. Interestingly, correction of the *AIFM1* variant significantly restored the structure and function of AIF, further improving the physiological state of patient-specific iPSC-derived neurons. This study demonstrates that the *AIFM1* variant is one of the molecular bases of ANSD. Mitochondrial dysfunction, especially _m_Ca^2+^ overload, plays a prominent role in ANSD associated with *AIFM1*. Our findings help elucidate the mechanism of ANSD and may lead to the provision of novel therapies.

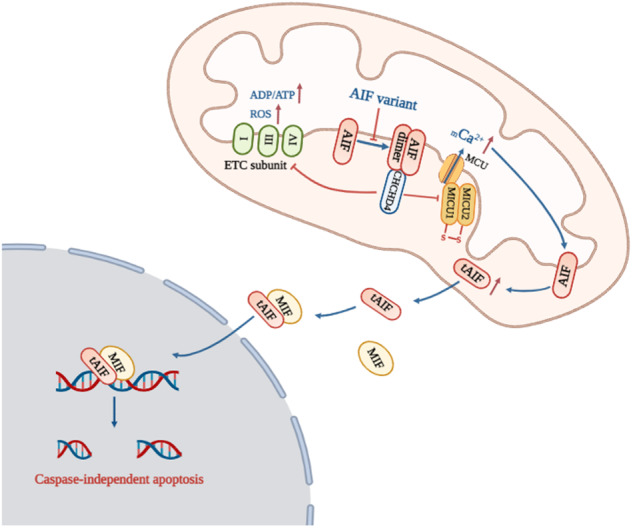

## Introduction

Auditory neuropathy spectrum disorder (ANSD) is a type of hearing impairment caused by the dysfunction of inner hair cells (IHCs), ribbon synapses, spiral ganglion neurons (SGNs) and/or the auditory nerve itself [1]. Approximately 1/7000 newborns have abnormal auditory nerve function, which accounts for 10%-14% of cases of permanent hearing loss in children [2–4]. Auditory neuropathy spectrum disorder is a major public health concern that places a substantial burden on families and society. Previous studies have identified several pathogenic genes for ANSD [5–12]; however, their mechanisms have remained poorly understood. We previously identified an ANSD pedigree with an *AIFM1* c.1265 G > A variant [13]. The patients presented with childhood-onset auditory neuropathy and delayed peripheral sensory neuropathy, with other symptoms including tinnitus, unsteadiness, and numbness in the extremities. The SGNs and auditory nerves were identified as lesion sites.

Apoptosis-inducing factor (AIF), encoded by *AIFM1*, is a flavoprotein located in the inner mitochondrial membrane space. It plays dual roles in cell death and survival [14–17]. AIF undergoes dimerization and conformational changes to form an air-stable FADH^•^-NAD^+^ charge transfer complex (CTC) [18–20]. AIF is present in a state of monomer-dimer equilibrium in mitochondria. The dimeric form enables essential mitochondrial functions by binding to CHCHD4 [21–23]. AIF knockdown in SGNs impaired mitochondrial respiration and disrupted membrane potential, indicating the vital role of AIF in mitochondrial functions [24]. Hearing loss has been identified among the symptoms of some known mitochondrial diseases, indicating that mitochondrial functions are crucial to auditory pathways [25–28]. These data have led to the recognition of an unrecognized role of AIF variants in the occurrence of ANSD via their regulatory effects on mitochondrial functions.

Mitochondria are one of the important calcium reservoirs and play a substantial role in calcium homeostasis [29]. Uncontrolled calcium uptake causes _m_Ca^2+^ overload, leading to mitochondrial oxidative stress [30] and cell apoptosis [31]. In addition, disruption of _m_Ca^2+^ homeostasis was associated with noise-induced hair cell loss [32] and pyridoxine-induced auditory neuropathy [33]. These data suggest an association between _m_Ca^2+^ disorder and the occurrence of ANSD. However, any potential mechanism through which AIF variants affect mitochondrial functions and contribute to ANSD has remained unclear due to a scarcity of suitable cell models.

Patient-specific iPSCs and their gene-corrected isogenic cell model have begun to provide a formidable tool for exploring the mechanisms of hearing loss [34–37]. Most such studies have utilized iPSC-derived IHCs as the experimental model. For example, patient-specific iPSCs carrying *MYO7A* [38] or *MYO15A* [39] variants were differentiated into IHCs. The effects of gene correction were then investigated. These studies revealed abnormal stereocilia assembly in patient cells. Another study demonstrated the impaired anion exchange in patient-specific iPSC-differentiated IHCs carrying *SLC26A4* variants [40]. However, it remains notable that such studies only offer preliminary explorations of the lesion phenotype, without determining the exact molecular mechanisms. Since AIF variants related to ANSD have been reported to affect the SGNs and auditory nerves [13], patient-specific iPSC-differentiated auditory neurons could be a valuable model for investigating the mechanisms of the occurrence of ANSD associated with *AIFM1*.

We previously used stably transfected cell lines to prove that AIF variants associated with ANSD (p.T260A, p.R422W, and p.R451Q) impair AIF dimerization, and NADH treatment improves cell survival by promoting AIF dimerization [41]. However, the downstream mechanisms triggered by impaired AIF dimerization remain unclear. In the present study, we obtained patient-specific iPSCs carrying *AIFM1* c.1265 G > A (p.R422Q) and achieved gene correction using the CRISPR/Cas9 system. Subsequently, control iPSCs (Con-iPSCs), patient-specific iPSCs (AN-iPSCs), and gene-corrected isogenic iPSCs (CORR-iPSCs) were differentiated into neurons to investigate the pathogenic mechanisms of auditory neuropathy spectrum disorder.

## Results

### Generation and gene correction of ANSD patient-iPSCs and differentiation into neurons

**Fig. 1:**
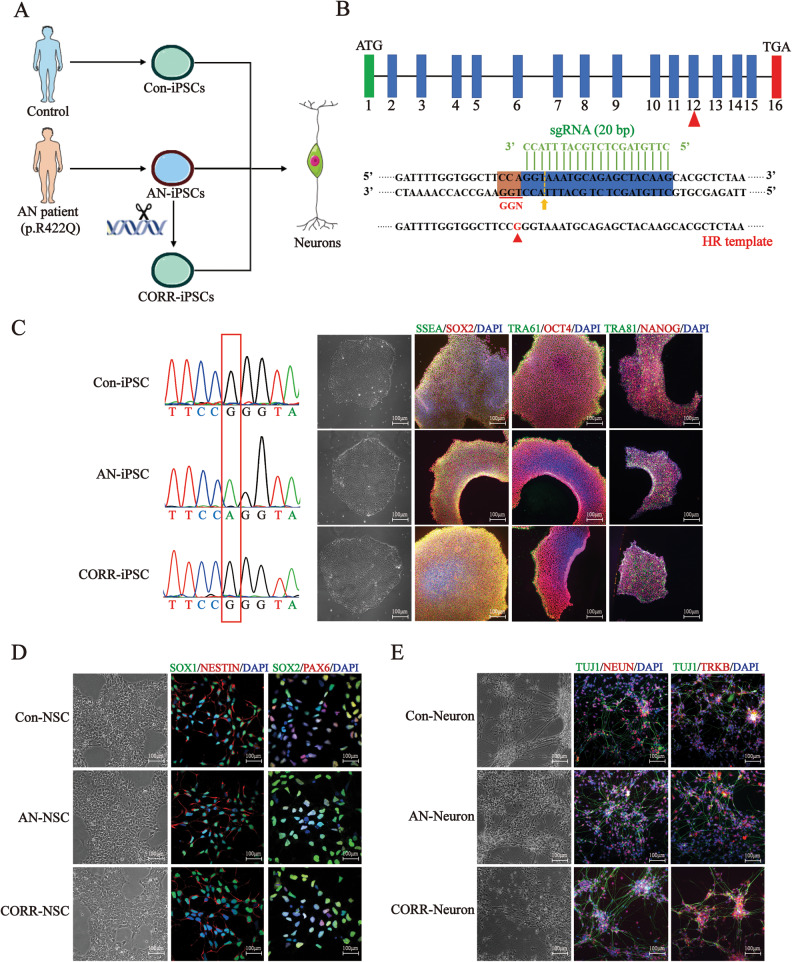
Generation and gene correction of ANSD patient-iPSCs and differentiation of neurons. **A** We generated iPSCs from PBMCs of both controls and patients. The AN-iPSCs were edited via CRISPR/Cas9 technology to generate the CORR-iPSCs. These iPSCs were further differentiated into neurons. **B** Sketch of the CRISPR/Cas9 system. The 20 bp sgRNA sequence is in green. The yellow arrow points to the cleavage site. The 150 bp ssDNA around the cleavage site was designed and synthesized as a homologous recombination template. **C** Identification of iPSCs. Left: *AIFM1* variant was present in AN-iPSCs but absent in Con-iPSCs and CORR-iPSCs. Right: Representative immunostaining for NANOG, SOX2, OCT4, TRA61, TRA81, and SSEA. Nuclei are stained blue with DAPI. **D** Identification of NSCs. Representative immunostaining for SOX1, SOX2, PAX6, and NESTEIN. Nuclei are stained blue with DAPI. **E** Identification of neurons. Representative immunostaining for TUJ1, NEUN, and TRKB. The mature neurons are stained with NEUN and TUJ1. The sensory neurons are stained with TRKB. Nuclei are stained blue with DAPI.

We collected PBMCs from the proband (III-8) and healthy control (IV-6) in the ANSD family (Fig. S1A). Sequencing results showed that the *AIFM1* c.1265 G > A variant was present in the ANSD patient but not in the control (Fig. S1B). We generated iPSCs from PBMCs by nucleofecting episomal plasmids. The AN-iPSCs were edited via CRISPR/Cas9 technology to generate the CORR-iPSCs. These iPSCs were further differentiated into neurons via NSCs (Fig. 1A). To generate the gene-corrected iPSCs, the optimized sgRNAs and homologous template were co-delivered into AN-iPSCs (Fig. 1B). To test our sgRNA and the cleavage efficiency of Cas9, the pX459-sgRNA plasmid was transfected into AN-iPSCs. The disarrayed sequencing peaks indicated that the sgRNA was efficient (Fig. S1C). We successfully obtained 2 edited clones out of 40 clones. The sequencing results revealed that the *AIFM1* variant was present in AN-iPSCs, but absent in Con-iPSCs and CORR-iPSCs (Fig. 1C). Immunofluorescence assays showed that all these iPSCs were stained with pluripotency markers, including NANOG, SOX2, OCT4, TRA61, TRA81, and SSEA (Fig. 1C). The qPCR assay also revealed the high expression of marker genes without significant differences in all three iPSC groups (Fig. S1D). Karyotype analysis also showed that the karyotype was normal (46, XY) (Fig. S1E). These iPSCs stained positively for alkaline phosphatase (Fig. S1F). Embryoid body (EB) assays generated cellular derivatives expressing ectoderm, mesoderm, and endoderm markers (Fig. S1G). All of these results indicated that high-quality iPSCs were successfully produced.

Subsequently, we identified NSCs and neurons with immunofluorescence, qPCR and western blotting. Immunofluorescence results showed that the NSCs were positive for specific markers, including SOX1, SOX2, PAX6, and NESTEIN (Fig. 1D), while the neurons also stained positively for neuron-specific markers, such as TUJ1, NEUN, and TRKB (Fig. 1E). The qPCR and western blotting results showed no significant differences in the differentiation efficiency of the three groups (Fig. S2A, S3A, S3B). These results indicated that we successfully generated neuron models for further investigation.

### The *AIFM1* c.1265 G > A mutation induced a splicing variant

**Fig. 2:**
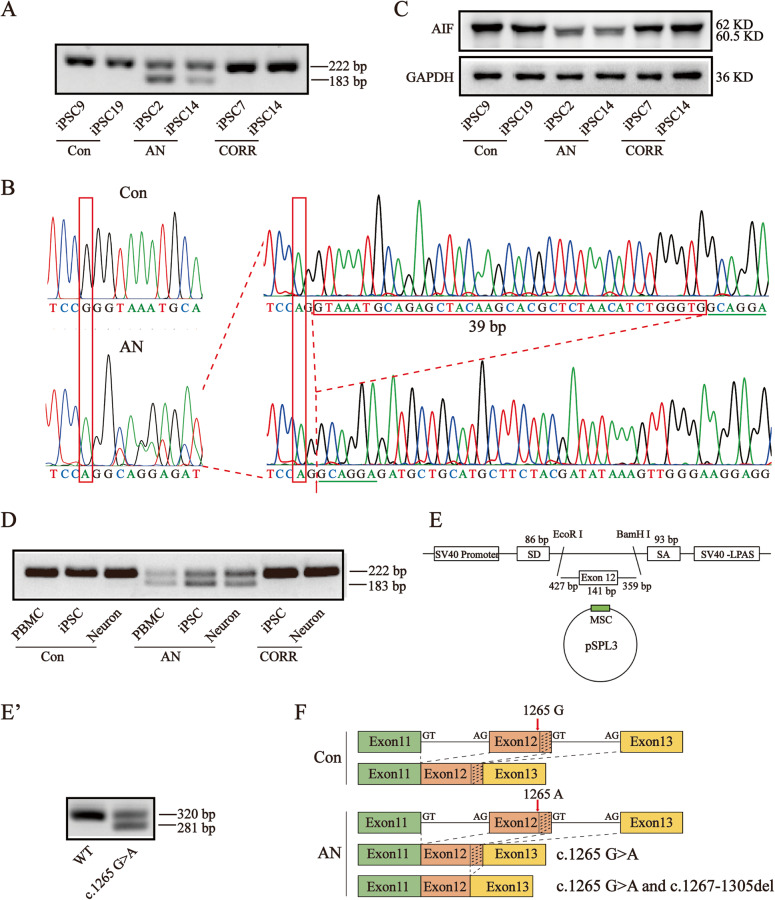
The *AIFM1* c.1265 G > A mutation induced the splicing variant. **A** Detection of the splicing variant of RNA from iPSCs via RT-PCR. The two bands were separated in agarose gel of RNA from AN-iPSCs. Forward primer: GAC GGC AGG AAG GTA GAA ACT. Reverse primer: AGC GTG ATC ATG GTG CTC TAC. Full-length gels are presented in Supplementary Fig. S7A. The cropped positions are labelled by red arrows. **B** Sequencing results of the two transcripts in AN-iPSCs. The *AIFM1* RNA sequence in AN-iPSCs presented as twin peaks, including a point mutation (c.1265 G > A) and a 39 bp deletion (c.1267-1305del). **C** Detection of the splicing variant in proteins from iPSCs via western blotting. The two bands were separated by SDS-PAGE of proteins from AN-iPSCs. Full-length blots are presented in Supplementary Fig. S7B. The cropped positions are labelled by red arrows. **D** Detection of the splicing variant of RNA from all cell types including PBMCs, iPSCs, and neurons, via RT-PCR. The splicing variant was present in all patient-specific cells. Full-length gels are presented in Supplementary Fig. S7C. The cropped positions are labelled by red arrows. **E**, **E'** The splicing variant was confirmed in vitro via an exon trapping system. The 927 bp gDNA including exon 12 and partial bases in intron 12 and intron 13 was amplified and constructed into a pSPL3 vector. RNA was extracted from 293 T cells after transfection with the vectors (pSPL3-*AIFM1*-WT/c.1265 G > A). RT-PCR and agarose gel electrophoresis were performed to test the splicing variant. Full-length gels are presented in Supplementary Fig. S7D. The cropped positions are labelled by red arrows. **F** The pattern diagram of RNA splicing. In control cells, RNA splicing was normal, producing a full-length *AIFM1* without a variant. In patient cells, RNA splicing was abnormal, generating an *AIFM1* mixture of the c.1265 G > A variant with the c.1267-1305del variant (with c.1265 G > A).

To identify the AIF variant at the transcriptional level, we cloned the *AIFM1* coding sequence (CDS). RT-PCR showed two bands in AN-iPSCs, but only one band in Con- and CORR-iPSCs (Fig. 2A). Disarrayed sequencing peaks were observed for AN-iPSCs. T-A cloning showed that the CDS was a mixture of point mutation with 39 base pair deletion (c.1267-1305del) transcripts (Fig. 2B). Consistently, western blotting showed two protein bands for AN-iPSCs, but only one protein band for Con-iPSCs and CORR-iPSCs at the translational level (Fig. 2C). These results indicated that the *AIFM1* c.1265 G > A mutation may induce a splicing variant in AN-iPSCs. RT-PCR was then performed for samples isolated from PBMCs, iPSCs, and neurons. We found that the splicing variant existed in all patient cells and could be rescued by correction of the point mutation (Fig. 2D). The splicing variant was determined to be patient-specific based on the results. Furthermore, the splicing variant was confirmed in vitro using the exon trapping system (Fig. 2E, E'). We further performed splicing prediction using “NetGene2” and “Splice Site Prediction by Neural Network” software, and the score of donor splice site 2 was increased from 0.79/0.96 to 0.86/0.99 with the AIF variant (Table S1). Collectively, these data suggested that the *AIFM1* c.1265 G > A mutation enhanced the splicing efficiency of another donor splice site, leading to the 39 bp deletion variant (Fig. 2F).

### AIF variants impaired AIF dimerization

**Fig. 3:**
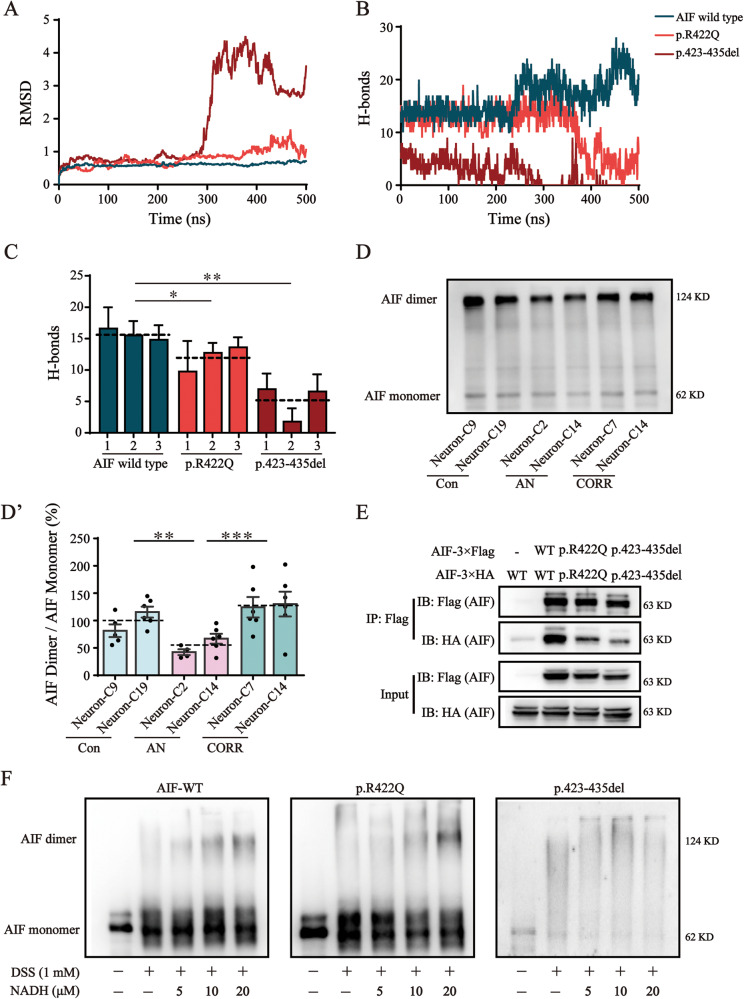
AIF variants impaired AIF dimerization. **A**, **B** Time-dependent RMSD values (**A**) and number of H-bonds (**B**) during MD simulations for wild type AIF and AIF variants. **C** The average numbers of H-bonds extracted from MD trajectories. Three independent MD simulations were performed. **D** Western blotting analysis of the levels of AIF dimer and AIF monomer in neurons after DSS-mediated cross-linking. The level of AIF monomers was used as a loading control. Full-length blots are presented in Supplementary Fig. S8A. The cropped positions are labelled by red arrows. **D'** Quantification analysis of the AIF dimer/AIF monomer. **E** The interaction between two AIF monomers was determined by Co-IP. Flag-tagged AIF and HA-tagged AIF were constructed and co-expressed in AIF-null cells. The interaction between two AIF variant monomers was weakened. Full-length blots are presented in Supplementary Fig. S8B. The cropped positions are labelled by red arrows. **F** NADH titration reduced AIF _oxidized_ into AIF _reduced_ in vitro. After purification, 1 μmol/L AIF was mixed with various concentrations of NADH (0 μmol/L, 5 μmol/L, 10 μmol/L, and 20 μmol/L). Then, 1 mmol/L DSS was added to cross-link the AIF dimer. Wild-type AIF could be reduced to a dimeric form using only a 5-fold excess of NADH, whereas AIF p.R422Q variant required 10-20-fold excess NADH for conversion to the reduced form. The AIF p.423-435del variant was refractory to reduction. Full-length blots are presented in Supplementary Fig. S8C. The cropped positions are labelled by red arrows. Data are represented as mean ± SEM and the value of Con-Neurons was normalized as 100%. *P < 0.05, **P < 0.01, ***P < 0.001.

The AIF p.422 residue locates in the dimerization interface. To measure the stability of the AIF dimer, MD simulations were performed. The results showed that the root mean square deviation (RMSD) in the atomic positions of the p.R422Q and p.423-435del variants fluctuated more widely than the AIF wild type (Fig. 3A). Hydrogen bond (H-bond) count analysis showed that the H-bonds at the contact surface had decreased along the MD trajectories in the p.R422Q and p.423-435del variant systems (Fig. 3B). Similarly, the average number of H-bonds in the p.R422Q and p.423-435del variants were also decreased compared to the AIF wild type (Fig. 3C). We analyzed the most stable snapshot from trajectories and found a decrease in the number of H-bonds along with an increase in the distance between two monomers of each AIF variant (Fig. S4B, S4C) compared to the AIF wild type (Fig. S4A). These results suggested that AIF dimerization may be impaired in the AIF p.R422Q and p.423-435del variants. Moreover, we investigated the content of AIF monomers and dimers in neurons and observed that AIF dimers were significantly reduced in AN-Neurons to 57.1% (*P* = 0.0014) of the corresponding value in Con-Neurons (Fig. 3D, D’). The interaction level between two monomers of the AIF p.R422Q variant and AIF p.423-435del variant was significantly decreased compared to that between the two monomers of wild-type AIF (Fig. 3E). Previous studies have reported that NADH binding primes AIF dimerization by reducing the oxidized state of AIF [18]. Therefore, we next mixed the purified AIF with NADH and discovered that wild-type AIF could be reduced to a dimeric form using only 5-fold excess NADH, whereas AIF p.R422Q variant required 10-20-fold excess NADH for conversion to the reduced form. The AIF p.423-435del variant was refractory to reduction (Fig. 3F). Concurrently, these data indicated that p.R422Q variant and p.423-435del variant affected AIF’s ability to bind with NADH, thus impairing AIF dimerization.

### AIF variants upregulated the ADP/ATP ratio and increased mitochondrial ROS levels

**Fig. 4:**
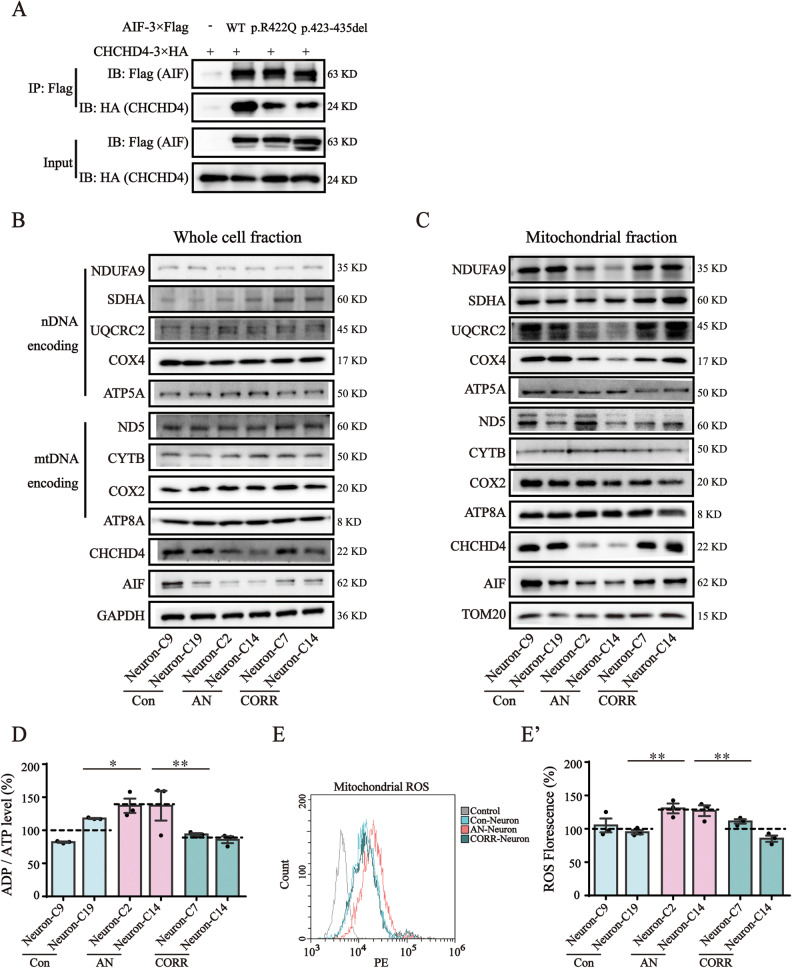
AIF variants upregulated the ADP/ATP ratio and increased mitochondrial ROS levels. **A** The interaction between AIF and CHCHD4 was determined by Co-IP. Flag-tagged AIF was co-expressed with HA-tagged CHCHD4 in AIF-null cells. The interaction between AIF variants and CHCHD4 was weakened. Full-length blots are presented in Supplementary Fig. S9A. The cropped positions are labelled by red arrows. **B** Western blotting analysis of the levels of AIF, CHCHD4, nDNA encoding ETC complex subunits (CI-NDUFA9, CII-SDHA, CIII-UQCRC2, CIV-COXIV, and CV-ATP5A), and mtDNA encoding ETC complex subunits (CI-ND5, CII-SDHA, CIII-CYTB, CIV-COX2, and CV-ATP8A) in the whole cell fraction. GAPDH is shown as a loading control. Full-length blots are presented in Supplementary Fig. S9B. The cropped positions are labelled by red arrows. **C** Western blotting analysis of the levels of AIF, CHCHD4, and nDNA/mtDNA coding ETC complex subunits in mitochondria. TOM20 is shown as a loading control. Full-length blots are presented in Supplementary Fig. S9C. The cropped positions are labelled by red arrows. **D** The ADP/ATP ratio was determined using a ADP/ATP Ratio Assay Kit. **E** Representative flow cytometry histogram overlay. Mitochondrial ROS was assessed using the fluorogenic dye MitoSOX™ Red reagent. **E'** Quantification analysis of the MitoSOX™ fluorescence intensity. The fluorescence intensity indicates the ROS level. Data are represented as mean ± SEM and the value of Con-Neurons was normalized as 100%. *P < 0.05, **P < 0.01.

CHCHD4 is an AIF binding partner with an important role in folding nuclear-encoded mitochondrial proteins within the IMS, including subunits of the electron transport chain (ETC) complex [42]. We performed a Co-IP assay to investigate the effect of AIF variants on the AIF-CHCHD4 interaction. The results revealed that the level of CHCHD4 pulled down by p.R422Q variant and p.423-435del variant was lower than that pulled down by wild-type AIF (Fig. 4A). To explore whether AIF variants affect the steady-state level and mitochondrial import of ETC subunits in AN-Neurons, the levels of these proteins in the whole cell fraction and mitochondrial fraction were measured. In the whole cell fraction, western blotting revealed that the levels of AIF and CHCHD4 were decreased, while there was no significant difference in the levels of ETC subunits encoded by either nuclear DNA (nDNA) or mitochondrial DNA (mtDNA) (Fig. 4B). In the mitochondrial fractions, the levels of ETC subunits encoded by nDNA, such as CI-NDUFA9, CIII-UQCRC2, and CIV-COX IV, in addition to AIF and CHCHD4 decreased markedly, but there were no significant differences in the levels of ETC subunits encoded by mtDNA (Fig. 4C). Furthermore, correction of the AIF variant restored the expression level and mitochondrial import of the above proteins (Fig. 4B, C). These results suggested that AIF variants lowered AIF protein levels, weakened the AIF-CHCHD4 interaction, destabilized CHCHD4 protein, and then inhibited mitochondrial import of ETC complex subunits encoded by nDNA without affecting their expression.

We next examined the ADP/ATP ratio and mitochondrial ROS level of Con-, AN-, and CORR-Neurons. The ADP/ATP ratio of AN-Neurons was ~14% (*P* = 0.022) higher than that of Con-Neurons; but 24% (*P* = 0.002) lower in CORR-Neurons than in AN-Neurons (Fig. 4D). The level of mitochondrial ROS in AN-Neurons was ~28.9% (*P* = 0.0027) higher than that in Con-Neurons (Fig. 4E, E’). We concluded that the AIF p.R422Q variant and p.423-435del variant inhibited the mitochondrial import of ETC complex subunits encoded by nDNA, subsequently leading to an upregulated ADP/ATP ratio and increased mitochondrial ROS levels.

### AIF variants induced mitochondrial calcium overload

**Fig. 5:**
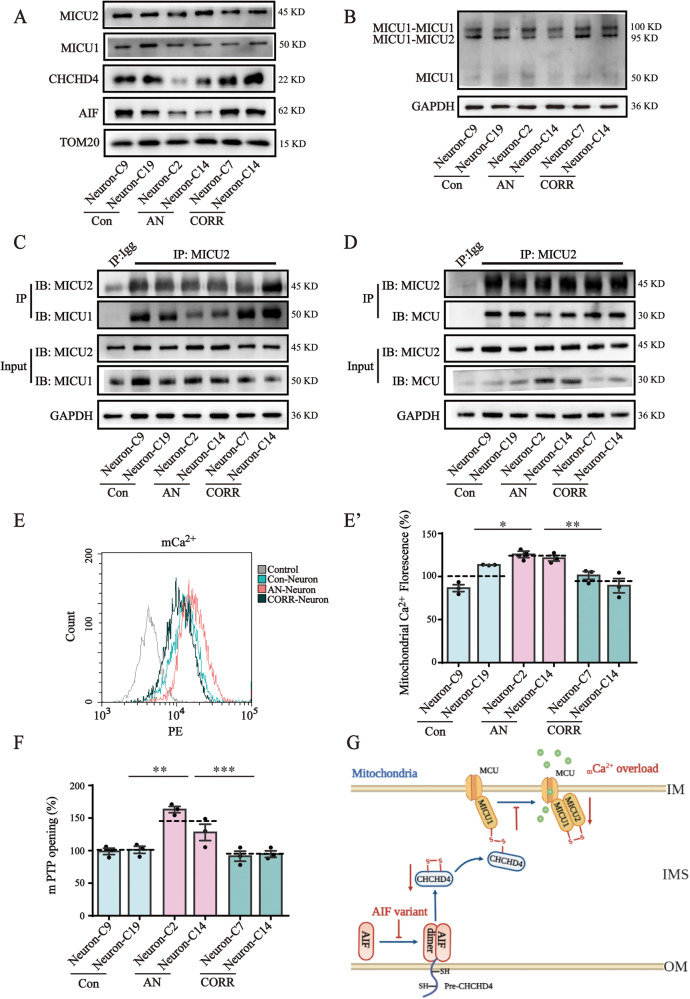
AIF variants induced mitochondrial calcium overload. **A** Western blotting analysis of the levels of AIF, CHCHD4, MICU1, and MICU2 in mitochondria. TOM20 is shown as a loading control. Full-length blots are presented in Supplementary Fig. S10A. The cropped positions are labelled by red arrows. **B** Western blotting analysis of the level of MICU1-MICU2 heterodimer in the absence of dithiothreitol (DTT). The level of MICU1-MICU2 heterodimers was decreased in AN-Neurons compared to that in Con- and CORR-Neurons. Full-length blots are presented in Supplementary Fig. S10B. The cropped positions are labelled by red arrows. **C** The interaction between MICU1 and MICU2 was detected using endogenous Co-IP. The MICU2 antibody was incubated with protein A/G agarose. The level of MICU1 pulled down by MICU2 indicated the strength of the MICU1-MICU2 interaction. Full-length blots are presented in Supplementary Fig. S10C. The cropped positions are labelled by red arrows. **D** The interaction between MCU and MICU2 was detected by endogenous Co-IP. The MICU2 antibody was incubated with protein A/G agarose. The level of MCU pulled down by MICU2 indicated the strength of the MICU2-MCU interaction. Full-length blots are presented in Supplementary Fig. S10D. The cropped positions are labelled by red arrows. **E** Representative flow cytometry histogram overlay. Mitochondrial calcium was assessed using Rhod2 reagent. **E'** Quantification analysis of the Rhod2 fluorescence intensity. The fluorescence intensity indicated the mitochondrial calcium level. **F** The opening of mPTP was measured using a Mitochondrial Permeability Transition Pore Assay Kit. The mean fluorescence intensity was analyzed using ImageJ software. **G** The mechanism of AIF variant-induced _m_Ca^2+^ overload. AIF variants inhibited AIF dimerization, resulting in a weakened AIF-CHCHD4 interaction. The mitochondrial import of CHCHD4 was then decreased. Subsequently, the MICU1-MICU2 heterodimer catalyzed by CHCHD4 was decreased, which disrupted the role of MICU2 in inhibiting MCU activity, causing _m_Ca^2+^ overload. The graph was drawn via the BioRender website (https://biorender.com/). Data are represented as mean ± SEM, and the value of Con-Neurons was normalized as 100%. *P < 0.05, **P < 0.01, ***P < 0.001.

Mitochondrial calcium uptake 1 (MICU1), a substrate of CHCHD4, participates in _m_Ca^2+^ uptake. CHCHD4 catalyzes MICU1-MICU2 heterodimer formation. MICU2 then interacts with MCU to inhibit _m_Ca^2+^ uptake [43]. To study the impacts of AIF variants on the _m_Ca^2+^ uniporter complex, we measured the protein levels of mitochondrial AIF, CHCHD4, MICU1, and MICU2 in Con-, AN-, and CORR-Neurons. AIF and CHCHD4 levels were significantly decreased in AN-Neurons but not in Con- or CORR-Neurons, while MICU1 and MICU2 levels were consistent and unchanged between these three groups (Fig. 5A). Intriguingly, the level of MICU1-MICU2 heterodimers was significantly downregulated in AN-Neurons compared to Con- and CORR-Neurons (Fig. 5B). In AN-Neurons, the interaction level between MICU1 and MICU2 was significantly reduced (Fig. 5C), and the interaction level between MICU2 and MCU was also weakened (Fig. 5D). These results imply that AIF variants impair the MICU1-MICU2 heterodimer, thereby hindering the interaction between MICU2 and MCU.

We further conducted _m_Ca^2+^ content assays. The _m_Ca^2+^ content was 23.5% (*P* = 0.0058) higher in AN-Neurons than in Con-Neurons, which then decreased to 95.4% (*P* < 0.0001) of that of Con-Neurons’ after gene correction (Fig. 5E, E’). The mPTP opening was also markedly increased by 45.5% (*P* = 0.0014) in AN-Neurons compared to Con-Neurons (Fig. 5F). Furthermore, we also found that AN-Neurons had reduced ER Ca^2+^ levels (Fig. S5A) and increased cytosolic Ca^2+^ levels (Fig. S5B). The increased cytosolic Ca^2+^ further upregulated MCU expression via the calmodulin/p-CaMKII/p-CREB pathway, as shown in Fig. S5C. Overall, AIF variants impaired MICU1-MICU2 heterodimerization, leading to _m_Ca^2+^ overload in AN-Neurons (Fig. 5G).

### AIF variants induced calpain-mediated AIF translocation and cell apoptosis

**Fig. 6:**
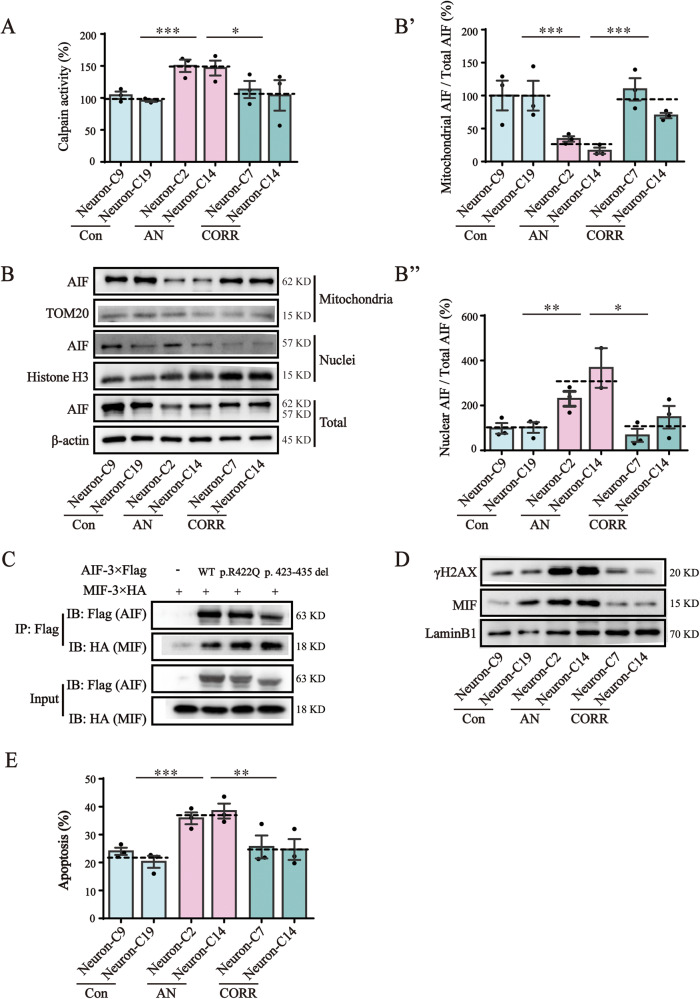
AIF variants induced calpain mediated AIF translocation and cell apoptosis. **A** Calpain activity was detected using a Calpain Activity Fluorometric Assay Kit. The results were read at 400 nm excitation and 505 nm emission. **B** Western blotting analysis of AIF localization via separating mitochondrial and nuclear fractions. The nuclear part is represented by H3 and the mitochondrial part is represented by TOM20. Full-length blots are presented in Supplementary Fig. S11A. The cropped positions are labelled by red arrows. **B'** Quantification analysis of the mitochondrial AIF/total AIF ratio. **B''** Quantification analysis of the nuclear AIF/total AIF ratio. **C** The interaction between AIF and MIF was determined by Co-IP. Flag-tagged AIF was co-expressed with HA-tagged MIF in AIF-null cells. The interaction between AIF variants and MIF was enhanced. Full-length blots are presented in Supplementary Fig. S11B. The cropped positions are labelled by red arrows. **D** Western blotting analysis of the levels of MIF and γH2AX in nuclei. LaminB1 was used as a loading control. Full-length blots are presented in Supplementary Fig. S11C. The cropped positions are labelled by red arrows. **E** Quantification analysis of apoptosis after flow cytometry detection. Cell apoptosis was tested using an Annexin V-FITC/PI Apoptosis Detection Kit. Data are represented as mean ± SEM, and the value of Con-Neurons was normalized as 100%. *P < 0.05, **P < 0.01, ***P < 0.001.

Mitochondria-localized calpains activated by _m_Ca^2+^ further induce AIF translocation. We found that the activity of calpains was ~1.5 times (*P* < 0.001) higher in AN-Neurons than in Con-Neurons (Fig. 6A). The mitochondrial AIF/total AIF ratio decreased significantly, while the nuclear AIF (tAIF)/total AIF ratio increased significantly (Fig. 6B, 6B’, 6B”) in AN-Neurons compared to Con- and CORR-Neurons. These results indicated that AIF variants provoked calpain activation, thereby increasing the importation of AIF from the mitochondria to the nuclei. We further analyzed the interaction between AIF and its related nucleases. The MIF proteins pulled down by the p.R422Q and p.423-435del variants were more abundant than that pulled down by wild-type AIF (Fig. 6C). Consistent with this, nuclear MIF levels were significantly increased in AN-Neurons relative to Con- and CORR-Neurons (Fig. 6D). We also observed the increased γH2AX expression in AN-Neurons, which is indicative of increased DNA damage (Fig. 6D). The apoptosis assay showed that AN-Neurons had a higher percentage of apoptotic cells (an average of 37.1%) compared to 22.1% (*P* < 0.001) in Con-Neurons and 25.1% (*P* = 0.002) in CORR-Neurons (Fig. 6E, S6A). Our results suggest that AIF variants recruited MIF into nuclei prior to causing cell apoptosis during calpain-mediated translocation of AIF. Furthermore, since cleaved-caspase expression did not differ significantly between Con-, AN-, and CORR-Neurons (Fig. S6B), we considered that a caspase-independent apoptosis pathway may be involved. Overall, our results demonstrated that calpain activation by _m_Ca^2+^ promoted AIF variants translocation into the nucleus, which ultimately induced apoptosis via a caspase-independent pathway.

## Discussion

In the current study, we generated patient-specific iPSCs and their derived gene corrected isogenic cell models. These iPSCs were further differentiated into neurons for mechanistic explorations of ANSD associated with *AIFM1* variants. The present study uncovered the mechanism underlying the upregulated ADP/ATP ratio, elevated mitochondrial ROS level, _m_Ca^2+^ overload, and subsequently activated apoptosis in patient-specific iPSC-differentiated neurons.

Patient-specific iPSCs, enabling the creation of gene-corrected isogenic cell models, provide a valuable tool for investigating the mechanisms of hearing loss. As previously reported, the diversity of genetic backgrounds in different donors may cause iPSC heterogeneity [44, 45]. This study rigorously explored the pathogenic mechanisms of AIF variants by correcting the *AIFM1* c.1265 G > A variant in AN-iPSCs. In this way, the influence of genetic background and age were excluded. We have shown that the original *AIFM1* c.1265 G > A variant was retained in AN-iPSCs but absent in Con- and CORR-iPSCs. The differentiated Con-, AN-, and CORR-Neurons strongly expressed neuron-specific marker genes, especially TRKB, BRN3A, and VGLUT2, which are considered auditory neuron-specific marker genes [46–48]. Previous studies have mostly utilized IHCs as the model for research, especially in hearing loss from IHCs damage [38, 39]. Another study differentiated iPSCs into CX26-gap junction plaque (GJP)-forming cells to investigate GJB2-related hearing loss from damage to CX26-GJP-forming cells [49]. A strong consideration for such studies is that the selection of the optimal cell type for differentiation depends on the lesion site. As previously reported, AIF variants tend to correlate with the impaired SGNs and auditory nerves in ANSD [13]. In the present study, auditory neurons were therefore more suitable for mechanistic investigations than inner hair cells. While relatively few studies have utilized iPSC-derived neurons to explore ANSD, there have been a few examples such as the testing of the toxic effect of quinine on the auditory system using iPSC-derived dopaminergic neurons [50] or the mechanistic exploration of Charcot-Marie-Tooth disease using iPSC-differentiated glial cells [51]. Here, we show AN-iPSC-differentiated auditory neurons as a positive model for exploring the mechanisms of ANSD associated with AIF variants.

Variants that affect splicing are significant contributors to disease [52]. This study identified a novel splicing variant in patient-specific iPSCs that occurs at both the transcriptional and translational levels. As previously reported, alternative splicing can occur during iPSC reprogramming and is vital in maintaining the pluripotent homeostasis of iPSCs [53]. One study indicated that a spliced form of CCNE1 significantly enhanced iPSCs reprogramming [54]. Another study demonstrated that RNA-binding proteins mediate considerable splicing alterations to promote somatic cell reprogramming [55]. To test whether the *AIFM1* splicing variant was introduced by iPSC reprogramming or was patient-specific, we analyzed the splicing variant in all patient samples, including PBMCs, iPSCs, and neurons. The splicing variant was proven to be patient-specific due to its existence in patient PBMCs. In fact, the majority of the reported mutations have been identified via genomic DNA sequencing. Therefore, it remained uncertain whether these point mutations could further cause splicing variants. *AIFM1* point variants have rarely been reported to induce splicing variants. One recent case report verified a novel intronic point variant of *AIFM1*, which caused abnormal RNA splicing and exon 11 skipping [56]. Presently, to determine whether the splicing variant was induced by the *AIFM1* c.1265 G > A variant, we analyzed the splicing variant in gene-corrected samples and performed exon capture assays. As expected, the splicing variant could be rescued after gene correction. Together, these results demonstrated that the *AIFM1* c.1265 G > A variant produced a splicing variant, which generated transcripts including both point and splicing variants. The functions of both point and splicing variants warrant further exploration. In actuality, we found that the splicing variant was easier to degrade, resulting in lowered AIF protein levels. AIF protein expression was essential for maintaining mitochondrial functions, as previously reported [57]. AIF knockdown in SGNs impairs mitochondrial respiration and disrupts the membrane potential [24]. Therefore, the elevated ADP/ATP ratio and increased ROS levels in our results may be partially due to the reduced AIF protein levels.

Mitochondrial calcium homeostasis is known to play essential roles in physiology and disease [29]. The disruption of mitochondrial calcium homeostasis has also been related to noise-induced loss of hair cells [32] and pyridoxine-induced auditory neuropathy [33]. However, the detailed mechanism behind such observations remains unclear. Our results demonstrate that the AIF-CHCHD4 interaction plays a crucial role in _m_Ca^2+^ regulation. AIF variants inhibited AIF dimerization, resulting in weakened AIF-CHCHD4 interactions. The mitochondrial import of CHCHD4 was then decreased, subsequently decreasing the MICU1-MICU2 heterodimer level and provoking _m_Ca^2+^ overload (Fig. 5G). Previous studies have indicated that AIF dimers are essential for the pulling of CHCHD4 into mitochondria [21, 22]. Here, we observed a reduced occurrence of the AIF dimeric form, weakening AIF-CHCHD4 interactions, and decreasing levels of mitochondrial CHCHD4 in AN-Neurons. These results demonstrate that AIF variants impaired AIF dimerization and subsequently inhibited CHCHD4 translocation into mitochondria. The level of MICU1-MICU2 heterodimers was also decreased, and the MICU1-MICU2 interaction was lessened in AN-Neurons. As shown in a previous report, CHCHD4 can oxidize MICU1 and introduce an intermolecular disulfide bond that links MICU1 with MICU2 [43]. We propose that AIF variants inhibit MICU1-MICU2 heterodimer formation predominantly due to the decreased CHCHD4 levels in mitochondria. Furthermore, our results also indicated a reduction in the MICU2-MCU interaction. MICU1 and MICU2 are mitochondrial calcium uptake regulatory proteins that sense calcium levels in the intermembrane space and regulate MCU-mediated _m_Ca^2+^ uptake [58]. Other studies have demonstrated that MICU2 inhibits MCU activity to prevent excessive _m_Ca^2+^ influx in a mechanism that is dependent on the presence of MICU1 [59, 60]. We next inferred that the weakened MICU2-MCU interactions were secondary consequences of decreased MICU1-MICU2 heterodimers. As expected, the _m_Ca^2+^ level was upregulated in AN-Neurons. Complementary to our findings, several studies have shown that a lack of MICU1-MICU2 heterodimers leads to increased _m_Ca^2+^ uptake [60–62]. A key role for mitochondrial calcium homeostasis in ANSD has also been reported previously [32, 33]; however, few studies have proceeded to investigate this clearly. Furthermore, no known research has expounded on any potential role of AIF in _m_Ca^2+^ regulation. In this study, we first proposed that AIF participates in _m_Ca^2+^ regulation via CHCHD4-mediated MICU1-MICU2 heterodimer formation and then deciphered the role of _m_Ca^2+^ in ANSD using patient-specific iPSC-derived neurons. The Ca^2+^ released from the ER is the main source of _m_Ca^2+^ influx [63]. Our study revealed that the levels of cytosolic Ca^2+^ and _m_Ca^2+^ increased, while the ER Ca^2+^ content decreased in AN-Neurons. We speculated that the excessive _m_Ca^2+^ influx caused by AIF variants may further induce ER Ca^2+^ release. In summary, our study involved constructing patient-specific iPSCs carrying the *AIFM1* c.1265 G > A variant and their gene-corrected isogenic models, and revealed that the *AIFM1* variant is one of the molecular bases of ANSD. Mitochondrial dysfunction, especially _m_Ca^2+^ overload, plays a prominent role in ANSD associated with AIF variants. The findings help elucidate the mechanism of ANSD and may lead to improved treatment of ANSD.

## Methods

### Subjects and clinical evaluations

The ANSD family with an *AIFM1* c.1265 G > A variant (Fig. S1A), which has been previously reported by our research group, was recruited in this study [13]. Written informed consent was obtained from all participants or from parents of child subjects in the family. All participants were examined with audiological tests and neurological examinations. The patients showed late-onset low frequency affected sensorineural hearing loss, impaired speech discrimination, cochlear nerve hypoplasia, and delayed peripheral sensory neuropathy [13]. The participants with normal hearing in the ANSD family were involved as normal controls. Here, PBMCs were isolated from patients and normal controls for further mechanistic explorations. The study was approved by the Committee of Medical Ethics of the Chinese PLA General Hospital (Approval No. of Ethics Committee: S2017-024-01). The study was registered at Chinese Clinical Trial Registry (registration number: ChiCTR2100050125).

### Generation of iPSCs

iPSCs were generated by nucleofection with episomal plasmids carrying OCT4, SOX2, KIF4, c-MYC, and LIN28. The detailed protocols are described in the Supplementary Materials. Pluripotent gene expression, karyotyping, alkaline phosphatase staining, and three germ layer differentiation assays were performed as reported previously [64]. All iPSCs were regularly tested for mycoplasma contamination.

### Gene correction mediated by CRISPR/Cas9

Single-guide RNAs (sgRNAs) were designed using the website http://crispor.tefor.net/crispor.py, and the ssDNA template was synthesized by GENEWIZ (Suzhou, China). The pX459-sgRNA and ssDNA template were co-delivered into AN-iPSCs by electroporation. The primers for off-target detection are shown in Table S2.

### Directed differentiation into auditory neurons

These iPSCs were further differentiated into auditory neurons via NSCs as previously reported [65]. The culture medium consists of neurobasal medium, 50×B-27 supplement, 100×GlutaMAX, 100×NEAA, 20 ng/ml BDNF, 20 ng/ml GDNF and 200 µmol/L L-AA. The detailed protocols are described in the Supplementary Materials.

### The Exon-Capture System for verifying splicing variants

The PCR fragments, with or without the *AIFM1* c.1265 G > A variant, were constructed into a pSPL3 vector (Invitrogen, California, USA). RNA was extracted from 293 T cells after transfection with each of the two plasmids. RT-PCR was performed to detect splicing variants. The related primers are listed in Table S3.

### Molecular dynamics simulation

The simulations were carried out using the GROMACS software package (version 2020.6) [66], together with the CHARMM36 force field set in explicit TIP3P water solvent. The conditions used for molecular dynamics (MD) are provided in the Supplementary Materials. The simulations were performed for 500 ns with three replicates. Trajectory analysis was performed in PyMOL.

### Cross-linking assay

Neurons were washed three times with ice-cold PBS (pH 8.0) to remove amine-containing culture media. Then, 4 mmol/L disuccinimidyl suberate (DSS) (Thermo Fisher Scientific, Rockford, USA) was used to cross-link the AIF dimer for 30 min at room temperature. Tris (20 mmol/L, Sangon, Shanghai, China) was used to quench the reaction for 15 min at room temperature.

### Co-Immunoprecipitation assay (Co-IP)

For exogenous Co-IP, the Flag-fused protein was incubated with Flag beads (Sigma-Aldrich, Missouri, USA) overnight at 4 °C. For endogenous Co-IP, the protein was incubated with MICU2 antibody (Absin, Shanghai, China) for 4 h. Next, protein A/G beads (Santa Cruz Biotechnology, California, USA) were added for overnight incubation. Washing was performed three times, and the beads were boiled for SDS-PAGE. The primers for exogenous Co-IP are listed in Table S4.

### Expression and purification of wild-type and variant AIF

The exogenously expressed proteins were extracted with lysis buffer. The protein supernatant was mixed with Flag beads (Sigma-Aldrich) and incubated at 4 °C for 6 h. After the beads were washed three times, 3×Flag Peptide (Sigma-Aldrich) was added for competitive elution of the recombinant protein. The primers for plasmid construction are listed in Table S4.

### NADH titration in vitro to reduce AIF _oxidized_ into AIF _reduced_

AIF (1 μmol/L) was mixed with various concentrations of NADH (0 μmol/L, 5 μmol/L, 10 μmol/L and 20 μmol/L) (Yeasen, Shanghai, China) in PBS buffer. Reactions were incubated for 15 min at room temperature to reduce AIF_oxidized_. Cross-linking was performed with 1 mmol/L DSS, followed by boiling with 1×SDS loading buffer. SDS-PAGE was then performed.

### Mitochondrial function assays

The ADP/ATP ratio was measured via an ADP/ATP Ratio Assay Kit (Sigma-Aldrich) according to the user’s manual. Mitochondrial ROS was assessed using the fluorogenic dye MitoSOX™ Red reagent (Invitrogen) according to the reported guidelines [67].

### Ca^2+^ assays

Mitochondrial Ca^2+^ was measured using a Rhod2-AM probe (4 μmol/L) (Invitrogen). Cytosolic Ca^2+^ was assayed via a Fluo4-AM probe (2 μmol/L) (Beyotime Biotechnology, Shanghai, China). Endoplasmic reticulum (ER) Ca^2+^ was measured using a Mag-Fluo-4 AM probe (5 μmol/L) (AAT Bioquest, California, USA). According to the user’s manual, fluorescence was detected in the PI and FITC channels via flow cytometry.

### mPTP opening assay

The opening of the mitochondrial permeability transition pore (mPTP) was measured using a Mitochondrial Permeability Transition Pore Assay Kit (Beyotime Biotechnology) according to the instructions. Photographs were taken with a fluorescence microscope at an excitation wavelength of 494 nm. The mean fluorescence intensity was analyzed using ImageJ software.

### Calpain activity and apoptosis assays

Calpain activity was detected using a Calpain Activity Fluorometric Assay Kit (BioVision, California, USA). The results were read at 400 nm excitation and 505 nm emission after incubation at 37 °C for 1 h in the dark. Cell apoptosis was tested using an Annexin V-FITC/PI Apoptosis Detection Kit (Yeasen). After staining, 1× binding buffer was added for flow cytometry.

### Nuclear and cytoplasmic separation

After the cells were broken using a Dounce homogenizer, differential centrifugation was used to separate the nuclear and mitochondrial fractions. The nuclear and heavy mitochondrial fractions were isolated from the supernatant via centrifugation at 1500 g for 10 min and at 10,000 g for 20 min, respectively. The detailed protocols are described in the Supplementary Materials.

### Western blotting assay and antibodies

The protein lysates were denatured and separated by 10% SDS-PAGE. The proteins were transferred to PVDF membranes (Millipore, Massachusetts, USA) and blocked with 5% milk for 1 h. The membranes were then incubated with the relevant primary and secondary antibodies. The source of antibodies (vendor and catalog number) are listed in Table S5.

### Immunofluorescence

Cells were fixed with 4% paraformaldehyde for 15 min and then permeabilized in 0.2% Triton® X-100 (Sigma-Aldrich) for 15 mins. After incubation with 5% BSA for 1 h at room temperature, the cells were stained with primary and secondary antibodies conjugated with fluorescein (Abcam, MA, USA). The primary antibodies are listed in Table S5.

### Quantitative real-time PCR

Total RNA was extracted with TRIzol reagent (Invitrogen). cDNA was obtained using a Prime Script™ RT Reagent Kit with gDNA Eraser (Takara, Osaka, Japan). Quantitative real-time PCR was performed using an ABI PRISM 7900HT Sequence Detection System (Applied Biosystems). β-actin was used as the reference gene for normalization. The primers are shown in Table S6.

### Statistical analysis

Statistical analyses were performed using GraphPad Prism software v.6.01. Two cell clones were selected for each group and quantitative data were obtained from at least three independent differentiation batches. The investigators were not blinded to allocation during experiments and outcome assessment. All data were tested for normal distribution and the variance was similar between the groups. No samples were excluded from the analyses performed. Statistical significance was analyzed using the two-tailed Student’s *t*-test. Most control groups were normalized to 100%. Data are represented as mean ± SEM. *P* < 0.05 was considered significant. *P < 0.05, **P < 0.01, ***P < 0.001.

## Supplementary information

Reproducibility checklist

Supplementary material

Uncropped full-length gels and blot

## Data Availability

All data generated or analyzed during this study are included in this published article and its Supplementary Information files. All data are available from the corresponding author on reasonable request.
