# Supplementary material for: Impaired AIF-CHCHD4 interaction and mitochondrial calcium overload contribute to auditory neuropathy spectrum disorder in patient-iPSC-derived neurons with AIFM1 variant
Source: Cell Death Dis. 2023 Jun 26;14(6):375. doi: 10.1038/s41419-023-05899-6 (PMC10293272; doi:10.1038/s41419-023-05899-6)
Supplement: Supplementary file 2 — Supplementary material [file 41419_2023_5899_MOESM2_ESM.docx]

**SUPPLEMENTARY MATERIALS**

**Impaired AIF-CHCHD4 interaction and mitochondrial calcium overload contribute to auditory neuropathy spectrum disorder in patient-iPSC-derived neurons with *AIFM1* variant**

**Running title: AIF variant causes mitochondrial Ca^2+^ overload in neurons**

Yue Qiu^1a^, Hongyang Wang^2a^, Mingjie Fan^1,3^, Huaye Pan^1^, Jing Guan^2^, Yangwei Jiang^1^, Zexiao Jia^1^, Kaiwen Wu^2^, Hui Zhou^1^, Qianqian Zhuang^1^, Zhaoying Lei^1^, Xue Ding^1^, Huajian Cai^1^, Yufei Dong^1^, Lei Yan^1^, Aifu Lin^1^, Yong Fu^4^, Dong Zhang^1^**^*^**, Qingfeng Yan^1,3,5b^**^*^**, and Qiuju Wang^2^**^*^**

^1^College of Life Sciences, Zhejiang University, Hangzhou, Zhejiang 310058, China

^2^Senior Department of Otolaryngology, Head and Neck Surgery, Chinese PLA Institute of Otolaryngology, Chinese PLA General Hospital, Beijing 100853, China.

^3^Department of Pediatrics, The First Affiliated Hospital of Zhejiang University School of Medicine, Hangzhou, Zhejiang 310003, China

^4^The Children’s Hospital of Zhejiang University School of Medicine, Hangzhou, Zhejiang 310052, China

^5^Key Laboratory for Cell and Gene Engineering of Zhejiang Province, Hangzhou, Zhejiang 310058, China

^a^ These authors contributed equally to this work.

^b^ Lead contact

**^*^Corresponding author:**

E-mail: Q.Y ([qfyan@zju.edu.cn](mailto:qfyan@zju.edu.cn)), D.Z ([zhangd_iqb@zju.edu.cn](mailto:zhangd_iqb@zju.edu.cn)), Q.W ([wqcr301@vip.sina.com](mailto:wqcr301@vip.sina.com) or [wqjavm301@sina.com](mailto:wqjavm301@sina.com) )

**SUPPLEMENTARY METHODS**

**Generation of iPSCs**

The iPSCs were generated by transfection with episomal plasmids including pCXLE-hOCT3/4 (Addgene, Massachusetts, USA, Cat. no. #27076), pCXLE-hSK (Addgene, Cat. no. #27078), and pCXLE-hUL (Addgene, Cat. no. #27080) via LONZA 4D (Maryland, USA). About 10 days after transfection, the clones were picked out. The iPSC clones were cultured in mTesR1 medium (Stem cell Technologies, Vancouver, Canada) and sub-cultured when reaching 70% confluence. The expression of the pluripotent genes was detected via immunofluorescence and Quantitative Real-Time PCR. Karyotyping, alkaline phosphatase staining, and three germ layers differentiation were performed as reported previously. Cells were harvested to detect the *AIFM1* c.1265G>A variant. The forward primer was: TAG GCA TAA ATG GAA ACT GTG G; and the reverse primer was: CAA TAA GTA GAA CCG GAA GAG C.

**Gene correction mediated by CRISPR/Cas9**

The single-guide RNAs were designed from website (http://crispor.tefor.net/crispor.py) and the ssDNA template was synthesized by GENEWIZ (Shanghai, China). We selected a single-guide RNA (sgRNA) with the highest efficiency (F: CTT GTA GCT CTG CAT TTA CC; R: GGT AAA TGC AGA GCT ACA AG). The sgRNA was constructed into the pX459 plasmid (Addgene). Then, the pX459-sgRNA and ssDNA template were co-delivered into AN-iPSCs by electroporation. After sequencing identification, the iPSC clones with corrected *AIFM1* gene were expanded and sub-cultured. The primers for off-target detection are shown in Table S2.

**Directed** **differentiation into auditory neurons**

These iPSCs were further differentiated into auditory neurons via NSCs. The starting density of iPSCs should be about 15–25% confluency before NSC induction. Then 2.5 mL NSC induction medium (Gibco, California, USA) was added every two days. On day7, NSCs (P0) were harvested. NSCs at passage 2-10 were further differentiated into neurons. NSCs were dissociate using Accutase (Stem cell Technologies) and sub-cultured onto laminin (Gibco)-coated dishes. The auditory neurons were generated after culture for 14 days with a medium consisting of neurobasal medium (Gibco), 50×B-27 (Gibco), 100×GlutaMAX (Gibco), 100×nonessential animal acids (Gibco), 20 ng/mL brain-derived neurotrophic factor (BDNF) (R&D, California, USA), 20 ng/mL glial cell-derived neurotrophic factor (GDNF) (R&D) and 200 µmol/L L-ascorbic acid (Sigma-Aldrich). The culture medium was changed every 2–3 days.

**Molecular dynamics simulation**

The structure of the two AIF monomer was generated via SWISS-MODEL server based respectively on the chain A and chain C of the reduced AIF complexed with NAD (PDB: 4BUR). The starting structure of AIF dimer was generated via PyMOL corresponding to the coordinates of AIF-4BUR. The FAD and NAD (A and B) ligands were then placed into the active site. The simulations were carried out with the GROMACS software package (version 2020.6), together with the CHARMM36 force field set in explicit TIP3P water solvent. The temperature is 300 K and the pressure is 1 bar. The long-range electrostatic interactions were analyzed via PME method and the van der Waals (vdW) interactions were calculated using a cutoff distance of 1.0 nm. The AIF dimers with ligands were solvated in a 12.5 Å x 12.5 Å x 12.5 Å water box with a 60310 TIP3P water model. The system was then neutralized with 187 sodium and 185 chloride ions in a normal saline concentration. The solvated system was firstly energy-minimized by 10,000 steps, followed by a 2500, 000 step equilibration (2 fs for each step).The simulations were performed for 500 ns in three replicates. The trajectory analysis was performed in PyMOL.

**Cross-linking assay**

Disuccinimidyl suberate (DSS) (Thermo Fisher Scientific), as a crosslinker, was used to stabilize the AIF dimer during denaturing gel electrophoresis. 1×10^6^ neurons were washed three times with ice-cold phosphate-buffered saline (PBS) (pH 8.0) to remove amine-containing culture media. The DSS solution was added to a final concentration of 4 mmol/L. The reaction mixture was incubated for 30 minutes at room temperature. The quench solution (20 mmol/L Tris) was added and incubated for 15 minutes at room temperature. Finally, the protein was extracted for further SDS-PAGE.

**Co-immunoprecipitation assay (Co-IP)** **exogenously**

After transfected with plasmids expressing tagged-fusion proteins, the cells were collected. The cell pellet was resuspended in lysis buffer containing 150 mmol/L NaCl, 50 mmol/L Tris-HCl (pH 7.4), 1 mmol/L EDTA, 0.5% NP-40, and cOmplete Tablets (Roche, New Naxi, USA). About 1.5 mg protein supernatant was incubated with 5 μL Flag beads (Sigma-Aldrich) overnight at 4 ℃. The beads were washed three times with wash buffer containing 300 mmol/L NaCl, 50 mmol/L Tris-HCl (pH 7.4), 5 mmol/L EDTA, and 0.5% NP40. After washing, the beads were boiled in loading buffer. The primers for plasmids construction are listed in Table S4.

**Expression and purification of wild-type and variant AIF**

To express wild-type and variant AIF in eukaryotic cells, the plasmids of p Flag/His-*AIFM1* were conducted. After transfecting and expressing in 293T cells, the protein was extracted with lysis buffer. About 30 mg protein supernatant was mixed with 60 μL Flag beads (Sigma) and incubated at 4 °C for 6 hours. After washing the beads three times with wash buffer containing 300 mmol/L NaCl, 60 μL 3×Flag Peptide (Sigma-Aldrich) was added to competitive elution of the recombinant protein overnight. A Bradford assay was performed to detect the concentration of purified protein. The primers for plasmid construction are listed in Table S4.

**Co-immunoprecipitation endogenously**

The protein A/G agaroses (Santa Cruz Biotechnology) were mixed with 5 μL MICU2 antibody (Absin) at 4 ℃ for 4 hours. 5×10^6^ neurons were collected for protein extraction. Then about 1.0 mg protein supernatant was incubated with protein A/G agaroses-MICU2 overnight at 4 ℃. The agaroses were washed three times with wash buffer and then boiled in loading buffer for further SDS-PAGE.

**ADP/ATP measurements**

The ADP/ATP ratio was measured using the ADP/ATP Ratio Assay Kit (Sigma-Aldrich). After removing culture medium, 90 μL ATP reagent was added to each well. Luminescence (RLUA) was read after incubation for 1 minute at room temperature. Another 10-minute incubation was required to deplete the ATP signal. The residual ATP signal was read as RLUB. Then, 5 μL ADP reagent was added to each well. After incubating for 1 minute, RLUC was read. The ADP/ATP ratio was calculated using the formula: (RLUC – RLUB) / (RLUA).

**Mitochondrial active oxygen detection**

Mitochondrial ROS was assessed using a fluorogenic dye-MitoSOX™ Red reagent (Invitrogen). The MitoSOX™ reagent was diluted to 5 μmol/L in HBSS buffer with Ca^2+^/Mg^2+^. Cells were incubated for 15 min at 37 °C, 5% CO_2_. The fluorescence was detected on PI channel using flow cytometry.

**Mitochondrial Ca^2+^ assay**

Mitochondrial Ca^2+^ was measured using Rhod2-AM probe (Invitrogen). About 50×10^4^ cells were collected. The cells were stained with 4 μmol/L Rhod2-AM regent in HBSS buffer without Ca^2+^ for 30 min at 37 °C, 5% CO_2_. After washing three times with PBS, the fluorescence was detected on PI channel using flow cytometry.

**ER Ca^2+^ assay**

ER calcium was measured using Mag-Fluo-4 AM probe (AAT Bioquest). Cells were stained with 5 μmol/L Mag-Fluo-4 AM regent in HBSS buffer without Ca^2+^ for 30 min at 37°C, 5% CO_2_, and washed three times with PBS. The fluorescence was detected on FITC channel using flow cytometry.

**Cytosolic Ca^2+^ assay**

Cytosolic calcium was assayed using Fluo-4 AM probe (Beyotime Biotechnology). Cells were stained with 2 μmol/L Fluo-4 AM regent in HBSS buffer without Ca^2+^ for 60 min at 37°C, 5% CO_2_, and washed three times with PBS. The fluorescence was detected on FITC channel via flow cytometry.

**Calpain activity assay**

The calpain activity was detected using Calpain Activity Fluorometric Assay Kit (BioVision). 1x10^6^ cell pellets were resuspended in 100 μL extraction buffer and incubated on ice for 20 minutes. After centrifuging at 10000 g for 1 min, the supernatant was transferred. The protein concentration was assayed and 100 μg protein was diluted in to 85 μL extraction buffer. Then, 10 μL 10X reaction buffer and 5 μL calpain substrate were added to each assay. Results were read in a 400 nm excitation and 505 nm emission after incubating at 37 °C for 1 hour in the dark.

**Nuclear and cytoplasmic separation**

The neurons were washed with ice-cold PBS, left on ice for 10 min and then resuspended in 500 µL isotonic homogenization buffer containing 250 mmol/L sucrose, 10 mmol/L KCl, 1.5 mmol/L MgCl_2_, 1 mmol/L Na-EDTA, 1 mmol/L Na-EGTA, 10 mmol/L Tris-HCl, pH 7.4 and 0.1 mmol/L phenylmethyl sulfonyl fluoride (PMSF). 100 µL cell suspension was centrifuged and lysed for whole cell protein detection. The remaining 400 µL cell suspension was transferred into Dounce homogenizer, and 40 strokes were needed to break the cells. The unbroken cells were spun down at 100 g for 5 min. The nuclear and heavy mitochondrial fractions were isolated from the supernatant via centrifugation at 1500 g for 10 min and 10,000 g for 20 min, respectively. The final supernatant was used as the cytosolic fraction. The nuclear fraction was washed three times with homogenization buffer containing 0.5% NP40.

**Apoptosis assay**

The cell apoptosis was tested using Annexin V-FITC/PI Apoptosis Detection Kit (Yeasen). The neurons were digested by Accutase without EDTA, and then centrifuged at 300 g for 5 min. The cells were washed twice with pre-cooled PBS and resuspended with 100 μL 1×binding buffer. Then, 5 μL Annexin V-FITC and 10 μL PI staining solution were added. After reacting at room temperature for 15 minutes, 400 μL 1×binding buffer was added for flow cytometer assay.

**Western blotting assay**

The protein lysates obtained from neurons were denatured and separated in 10% sodium dodecyl sulfate polyacrylamide gels (SDS-PAGE). The proteins were then transferred to the PVDF membranes and blocked with tris-buffered saline and Tween 20 (TBST) (150 mmol/L NaCl, 10 mmol/L Tris-HCl, pH 7.5 and 0.1% (v/v) Tween 20) containing 5% milk for 1 hour. The membranes were then incubated with the relevant primary and secondary antibodies. The primary antibodies are listed in Table S5.

**Immunofluorescence**

Cells were fixed with 4% paraformaldehyde for 15 min and then permeabilized in 0.2% Triton® X-100 (Sigma-Aldrich) for 15 mins. After incubating with 5% BSA for 1 hour at room temperature, the cells were stained with primary and secondary antibodies conjugated with fluorescein (Abcam). Fluorescence intensities were obtained using a confocal microscope (FV3000) and were analyzed using the FV31S analysis software. The primary antibodies are listed in Table S5.

**Quantitative Real-Time PCR**

The total RNA was extracted with Trizol reagent (Invitrogen). cDNA was obtained using a Prime Script™ RT reagent Kit with gDNA Eraser (Takara). Quantitative real-time PCR for expression of pluripotent genes (NANOG, SOX2, TEX1, and OCT4), ectoderm markers (PAX6 and MAP2), mesoderm markers (TBX1 and MSX1), endoderm markers (SOX17 and AFP), NSC-specific markers (SOX1, SOX2, PAX6, and NESTIN) and neuron-specific markers (NEUN, TUJ1, BRN3A, TRKB, MAP2, and VGLUT2) was performed on an ABI PRISM 7900HT Sequence Detection System (Applied Biosystems). β-actin is used as the reference gene for normalization. The forward and reverse primers for PCR amplification are shown in Table S6.

**SUPLEMRNTAL FIGURES**


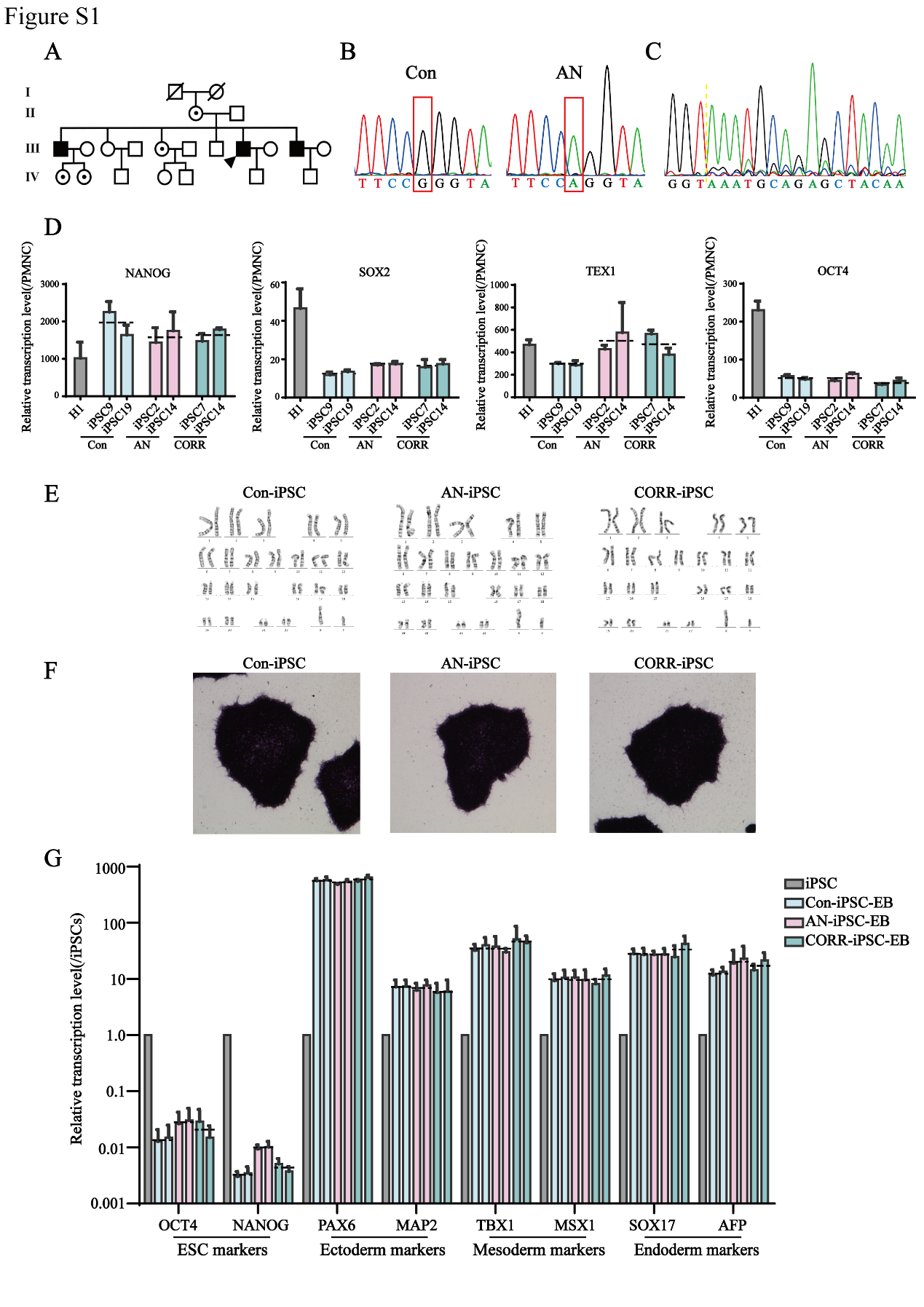


**Figure S1. Generation and identification of iPSCs.** (A) The pedigree of ANSD family. The patients were filled black and the proband was pointed out by arrow. (B) Identification of the *AIFM1* c.1265G>A variant in ANSD patient. The sequencing results showed the *AIFM1* c.1265G>A was present in patient but absent in control. (C) The efficiency of the sgRNA. The disarrayed sequencing peaks indicated the sgRNA is efficient. (D) The expression of iPSCs-specific marker. These iPSCs highly expressed NANOG, SOX2, TEX1, and OCT4. The PMNC was shown as a control cell. (E) Karyotype analysis of iPSCs. These iPSCs had a normal karyotype (46, XY). (F) Alkaline phosphatase assay. These iPSCs were positively stained for alkaline phosphatase. (G) The potential to differentiate into three germ layer. These iPSCs generated cellular derivatives expressing ectoderm markers (PAX6 and MAP2), mesoderm markers (TBX1 and MSX1), and endoderm markers (SOX17 and AFP). And the expression of pluripotent markers (OCT4 and NANOG) was decreased. The iPSC was shown as a control cell. Data are represented as mean ± SEM. **P*<0.05, ***P*<0.01, ****P*<0.001.


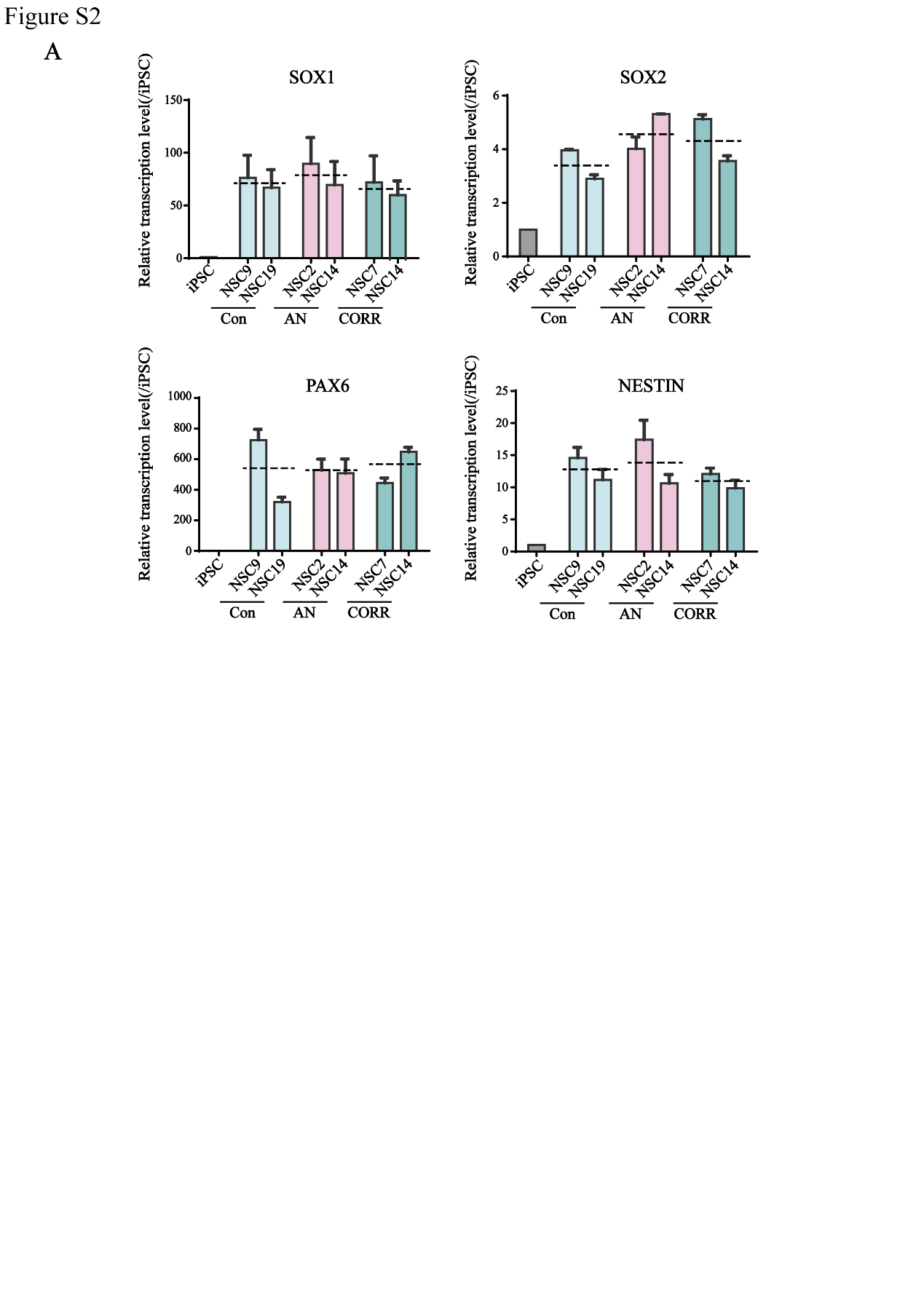


**Figure S2. Generation and identification of NSCs.** (A) The expression of NSCs-specific marker. These NSCs highly expressed SOX1, SOX2, PAX6, and NESTEIN. The iPSC was shown as a control cell. Data are represented as mean ± SEM. **P*<0.05, ***P*<0.01, ****P*<0.001.


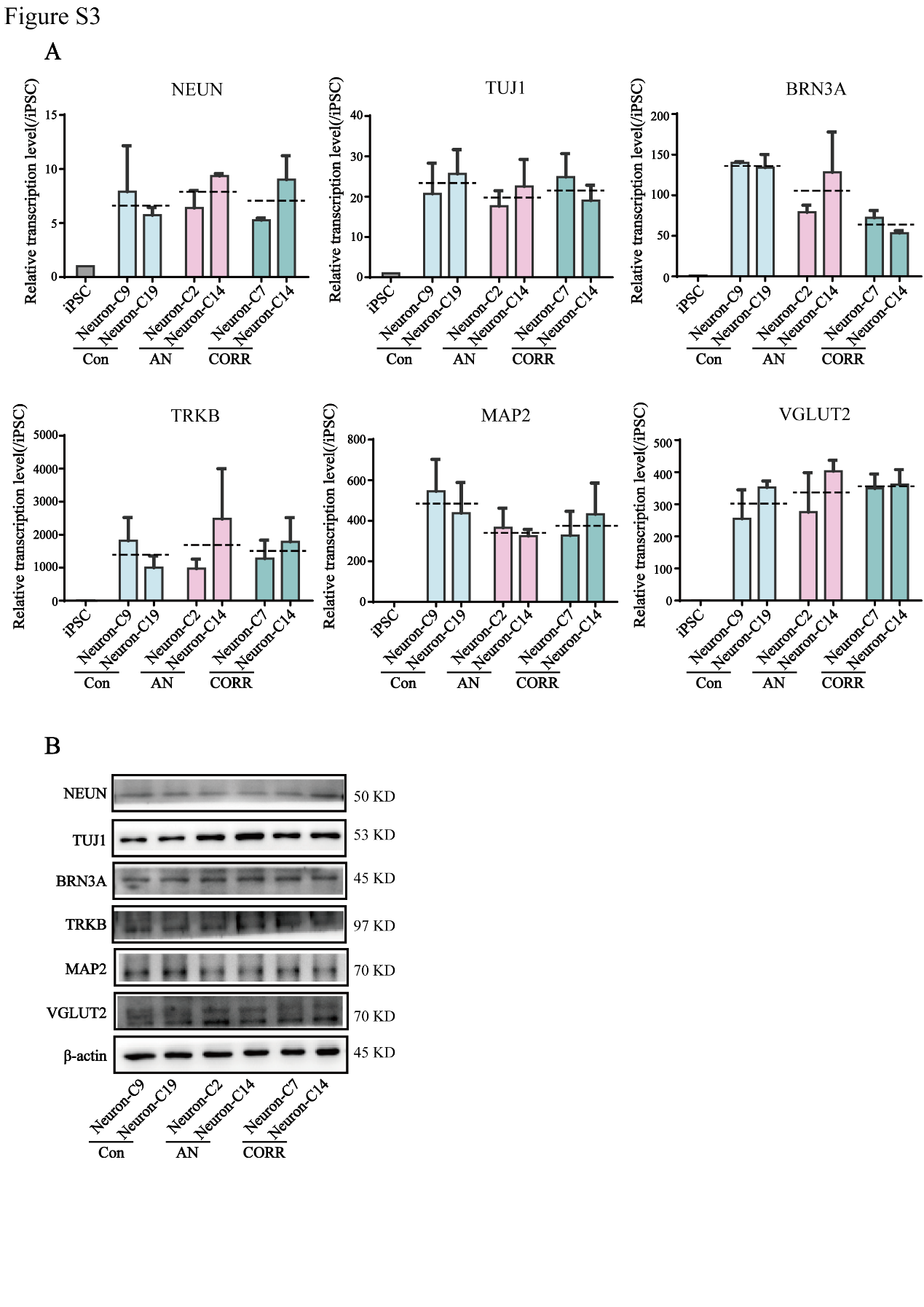


**Figure S3. Generation and identification of Neurons.** (A) The expression of neurons-specific marker gene. These neurons highly expressed NEUN, TUJ1, BRN3A, TRKB, MAP2, and VGLUT2. The iPSC was shown as a control cell. (B) The expression of neurons-specific marker protein. Western blotting analysis showed these neurons highly expressed NEUN, TUJ1, BRN3A, TRKB, MAP2, and VGLUT2. β-actin is used as the reference gene for normalization. Full-length blots are presented in Supplementary Figure S12. The cropped positions were labelled by red arrows. Data are represented as mean ± SEM. **P*<0.05, ***P*<0.01, ****P*<0.001.


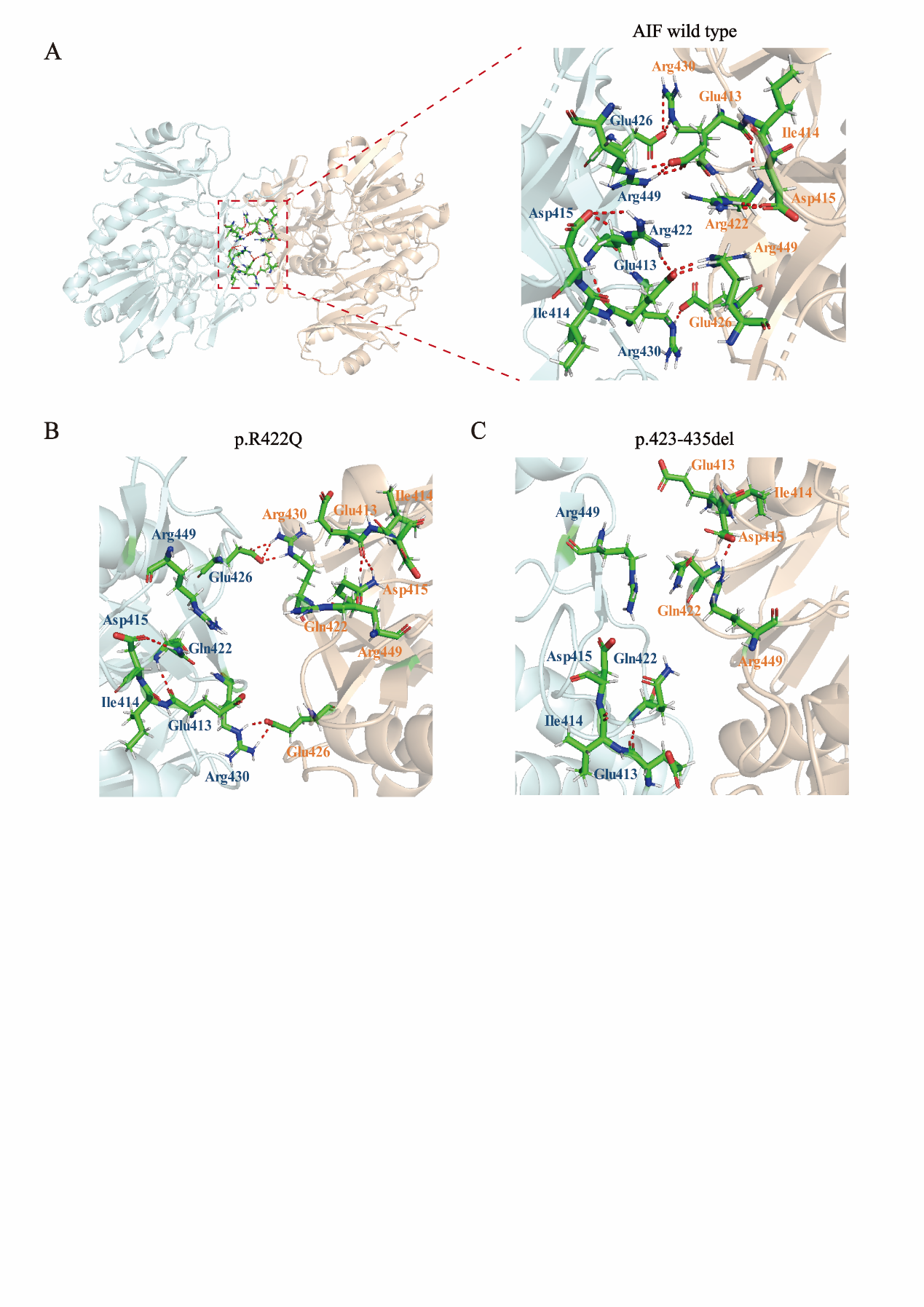


**Figure S4. The H-bonds at AIF dimer interface.** The dimer structure of (A) AIF wild type, (B) AIF p.R422Q variant and (C) AIF p.423-435del variant. The residues are colored by element. The C atom is in green, H atom is in silver, N atom is in blue, O atom is in red, and S atom is in yellow. Dashed red lines represent hydrogen bonds.


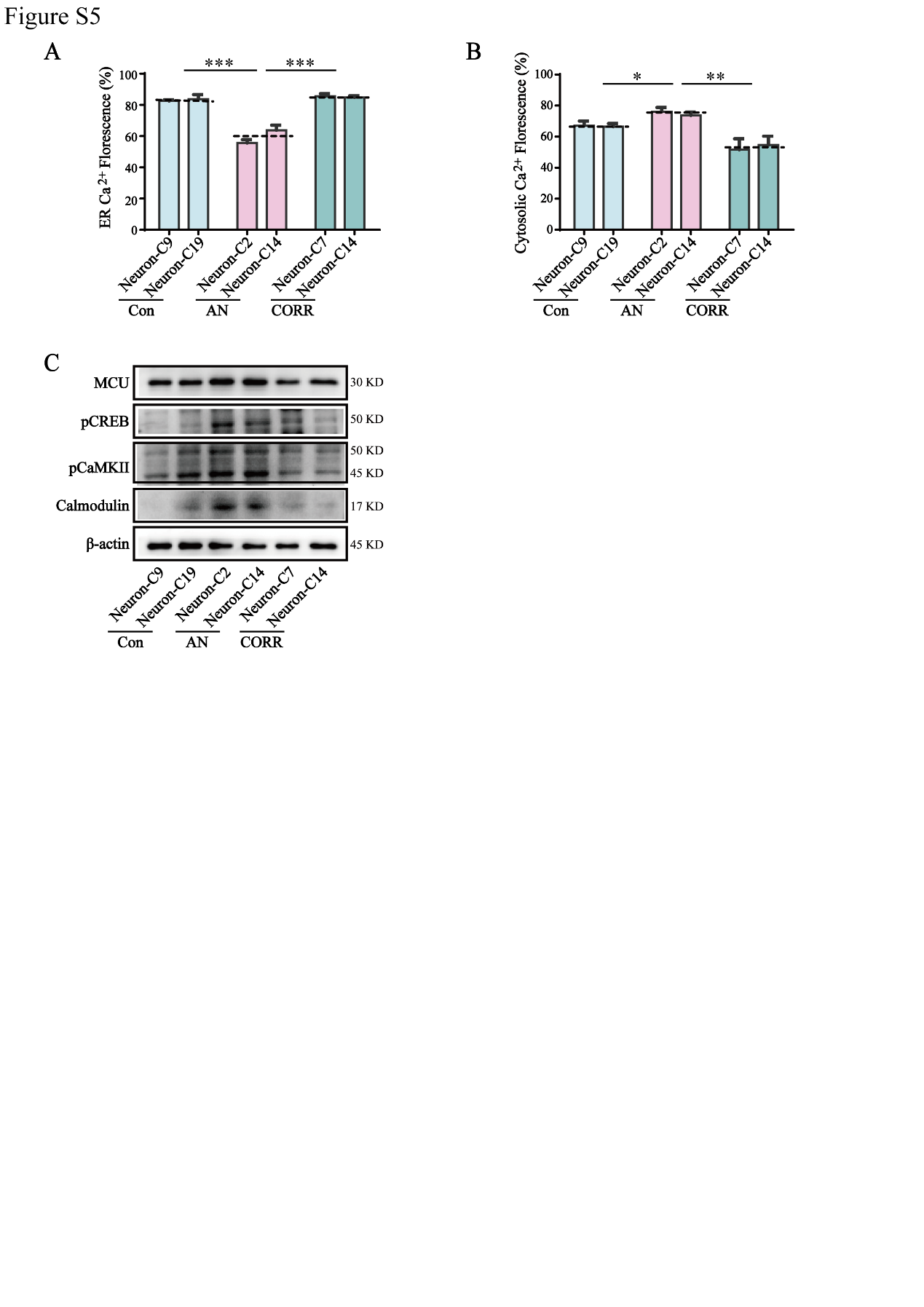


**Figure S5. Decreased** **ER calcium and increased cytosolic calcium in AN-Neurons.** (A) Quantification analysis for the ER calcium. The ER Ca^2+^ was measured using 5 μmol/L Mag-Fluo-4 AM probe (AAT Bioquest). (B) Quantification analysis for the cytosolic calcium. (C) Western blotting for MCU, p-CREB, p-CaMKII, and calmodulin. β-actin is used as the reference gene for normalization. Full-length blots are presented in Supplementary Figure S13. The cropped positions were labelled by red arrows. Data are represented as mean ± SEM. **P*<0.05, ***P*<0.01, ****P*<0.001.


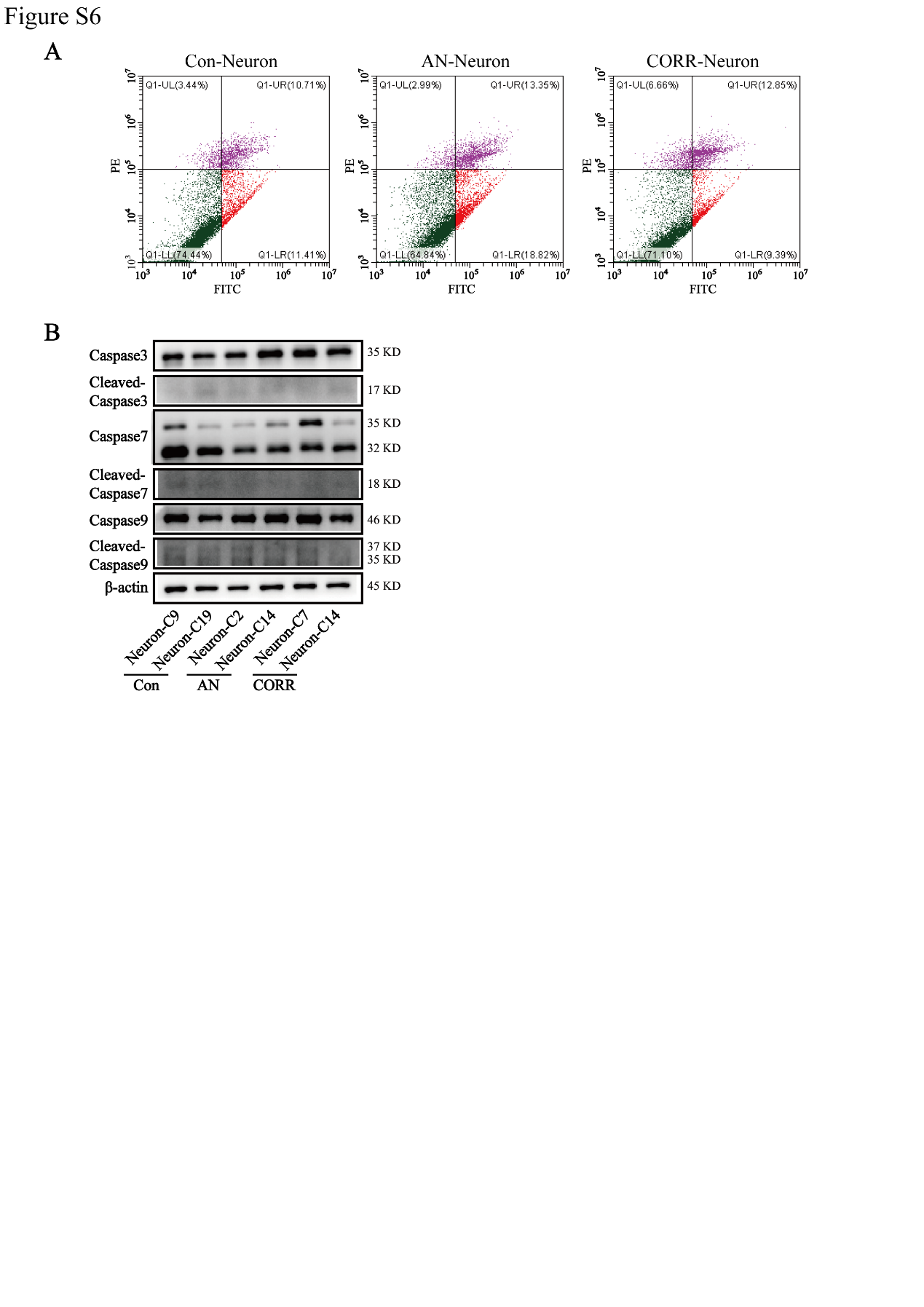


**Figure S6. The increased apoptosis was caspase-independent.** (A) Representative flow cytometry dot-plots of apoptosis. (B) Western blotting for Caspase3, 7, and 9. β-actin is used as the reference gene for normalization. Full-length blots are presented in Supplementary Figure S14. The cropped positions were labelled by red arrows.

**SUPLEMRNTAL TABLES**

Table S1. The efficiency of the splice sites were predicted via software.

Prediction results of “NetGene2”

| NetGene2 |  | Sequence | Score |
| --- | --- | --- | --- |
| WT | Donor site 1 | tgggtg gt aagtg | 1.00 |
|  | Donor site 2 | ttccg^a^g gt aaatg | 0.79 |
|  | Accept site 1 | tgcctc ag gtaga | 1.00 |
| c.1265 G>A | Donor site 1 | tgggtg gt aagtg | 1.00 |
|  | Donor site 2 | ttcca^b^g gt aaatg | 0.86 |
|  | Accept site 1 | tgcctc ag gtaga | 1.00 |

Prediction results of “Splice Site Prediction by Neural Network”

| Splice Site Prediction by Neural Network | |  | Sequence | Score |
| --- | --- | --- | --- | --- |
| WT | Donor site 1 | | tgggtg gt aagtg | 0.99 |
|  | Donor site 2 | | ttccg^a^g gt aaatg | 0.96 |
|  | Accept site 1 | | tgcctc ag gtaga | 1.00 |
| c.1265 G>A | Donor site 1 | | tgggtg gt aagtg | 0.99 |
|  | Donor site 2 | | ttcca^b^g gt aaatg | 0.99 |
|  | Accept site 1 | | tgcctc ag gtaga | 1.00 |

The splicing scores of splice sites were predicted via “NetGene2” and “Splice Site Prediction by Neural Network” software. The closer the score is to 1, the probability of splicing is higher. ^a^ : The “g” in wildtype *AIFM1* was in green. ^b^ : The “a” in mutant *AIFM1* was in red.

Table S2. The primers for off-target detection.

|  |  | Sequence (5’→3’) |
| --- | --- | --- |
| Off-target-1 | Forword | AGAAACAAGAATCCCCTTAA |
|  | Reverse | AGCAGAGGCTGGAACTGA |
| Off-target-2 | Forword | TAAATGTGCCATAGTCCA |
|  | Reverse | CAGGTGCAAATTCCTAAA |
| Off-target-3 | Forword | TAGTCTACCTTCACGCAA |
|  | Reverse | AACGGATATTTACAAACTTC |
| Off-target-4 | Forword | ATTGCTGGTGACTGGAAC |
|  | Reverse | ATAACTCACTGGCCTGTTT |
| Off-target-5 | Forword | TCATCGGCAGTCCTCAAC |
|  | Reverse | GGACGCCTGATGCTTCTC |
| Off-target-6 | Forword | TCCAGGAATAAATAAGGTTG |
|  | Reverse | CCTATGAAATAGCGAGCA |
| Off-target-7 | Forword | GGCTCGACTGGAGTGTTC |
|  | Reverse | CACCTTAGGAGGGCAACC |
| Off-target-8 | Forword | CTGAAGGCTTAGGACTGTTG |
|  | Reverse | TCTCCCAGGCATCTTTCTC |
| Off-target-9 | Forword | TTAATTCCCTTTGGTAGATG |
|  | Reverse | AGAGGTCCTCCTTCAGCT |
| Off-target-10 | Forword | AAGTGGCTTCATTCATAA |
|  | Reverse | TTCTCCTAAGTTTTGTGG |
| Off-target-11 | Forword | CAAGCATTCACAAGAAGT |
|  | Reverse | GTCTCAAAACTAGGCTGA |
| Off-target-12 | Forword | CTGTGAATCATTTCGGATAA |
|  | Reverse | GGACCCAGCTACCTCTAC |
| Off-target-13 | Forword | TCAATCTAGTGGGCATAAA |
|  | Reverse | GGCCTAAATCCCTACTTC |
| Off-target-14 | Forword | ACAGCCATCTGAGACAAA |
|  | Reverse | TTTCCAACATCCCAAGAG |
| Off-target-15 | Forword | CTTCCCAATTATTAACCTTC |
|  | Reverse | TACATCCTCCAATGACTT |
| Off-target-16 | Forword | AGAGGCGCTCAGAGTAAA |
|  | Reverse | AGAGCCACTTCTCGTTCC |
| Off-target-17 | Forword | ATGTTTCAAGGGAGGGTGGC |
|  | Reverse | CGGCCAGTGTCCCATATCTT |
| Off-target-18 | Forword | AGGCAGGTATCAGAGGCAGA |
|  | Reverse | CCACACAGTCTAGCTGGCAA |
| Off-target-19 | Forword | AGGTGGTGACTCTGCTTA |
|  | Reverse | CTGATTACTTATTTGGTC |
| Off-target-20 | Forword | ACTCGCCAGCATACCAGC |
|  | Reverse | AAGGGACTGAGCACTTTGG |

Table S3. Primers for Exon-Capture System construction.

|  |  | Sequence (5’→3’) |
| --- | --- | --- |
| pSPL3-*AIFM1* | Forword | GTACGGGATCACCAGAATTCGCCTTCCAGTTCTCCACTCC |
|  | Reverse | TCACCAGATATCTGGGATCCGCTTCATCCTCCATCCTCCG |
| Splice Test | Forword | TCACCTGGACAACCTCAAAG |
|  | Reverse | GAATTGGTCGAAATGGATC |

Table S4. Primers for vector construction.

|  |  | Sequence (5’→3’) |
| --- | --- | --- |
| p3×Flag-*AIFM1* | Forword | TTAAGCTTGCGGCCGCGAATTCATGTTCCGGTGTGGAGGCCTG |
|  | Reverse | TCCTCTAGAGTCGACTGGTACCGTCTTCATGAATGTTGAATA |
| p3×HA-*AIFM1* | Forword | ATTGAATTCCCCGGGGATCCATGTTCCGGTGTGGAGGCCTG |
|  | Reverse | GTATGGGTAGTCGACTCTAGAGTCTTCATGAATGTTGAATA |
| p3×HA-*CHCHD4* | Forword | ATTGAATTCCCCGGGGATCCATGTCCTATTGCCGGCAGGAAG |
|  | Reverse | GTATGGGTAGTCGACTCTAGAACTTGATCCCTCCTCTTCTTTGG |
| p3×HA-*MIF* | Forword | ATTGAATTCCCCGGGGATCCATGCCGATGTTCATCGTAAACACC |
|  | Reverse | GTATGGGTAGTCGACTCTAGAGGCGAAGGTGGAGTTGTTCCA |
| pFlag/His-*AIFM1* | Forword | GCTAGCGCCACCATGGCGGCCGCAATGTTCCGGTGTGGAGGCCT |
|  | Reverse | TCACCGGTAAGCTTTGCGATCGC GTCTTCATGAATGTTGAATA |

Table S5. Antibody used for western blot.

| \| Antibody \|  \|  \| \| --- \| --- \| --- \| | Company | Lot |
| --- | --- | --- | --- | --- | --- |
| Anti-AIF | Santa Cruz | ab32516 |
| Anti-Flag | Affinity | T0053 |
| Anti-HA | CST | #2367 |
| Anti-NDUFA9 | Affinity | DF4206 |
| Anti-SDHA | Affinity | DF7043 |
| Anti-UQCRC2 | diagbio | db2198 |
| Anti-COX4 | diagbio | db15 |
| Anti-ATP5A | diagbio | db4393 |
| Anti-TOM20 | AB clonal | A19403 |
| Anti-ND5 | Abcam | ab92624 |
| Anti-CYTB | Sigma-Aldrich | AV50256 |
| Anti-COX2 | Abcam | ab79393 |
| Anti-ATP8A | Santa Cruz | sc-84231 |
| Anti-MICU1 | absin | abs149728 |
| Anti-MICU2 | absin | abs151357 |
| Anti-CHCHD4 | Protein tech | 21090-1-AP |
| Anti-GAPDH | BK | BK7021 |
| Anti-MCU | Abcam | ab272488 |
| Anti-Histone H3 | Affinity | AF0863 |
| Anti-ß-actin | BK | BK7018 |
| Anti-CYPA | Protein tech | 10720-1-AP |
| Anti-γH2AX | Abcam | ab81299 |
| Anti-MIF | diagbio | db5845 |
| Anti-LaminB1 | Affinity | AF5161 |
| Anti-NEUN | Abcam | ab177487 |
| Anti-TUJ1 | Abcam | ab18207 |
| Anti-BRN3A | Affinity | DF4771 |
| Anti-TRKB | Affinity | AF6461 |
| Anti-MAP2 | Abcam | ab18207 |
| Anti-VGLUT2 | CST | #14487 |
| Anti-SOD2 | Protein tech | 24127-1-AP |
| Anti-CATALASE | Protein tech | 21260-1-AP |
| Anti-GPX5 | Protein tech | 18731-1-AP |
| Anti-PRDX6 | Abcam | ab133348 |
| Anti-p CREB | Abcam | ab32096 |
| Anti-p CaMKII (Thr287) | Affinity | AF3434 |
| Anti-p CaMKII (Thr286) | Abcam | ab124880 |
| Anti-Calmodulin | Abcam | ab45689 |
| Anti-Caspase3 | CST | #9662 |
| Anti-Caspase7 | CST | #12827 |
| Anti-Caspase9 | CST | #9502 |

Table S6. Primers for Quantitative Real-Time PCR.

|  |  | Sequence (5’→3’) | |
| --- | --- | --- | --- |
| ß-actin | Forword | | AGAAAATCTGGCACCACACCT |
|  | Reverse | | GATAGCACAGCCTGGATAGCAA |
| SOX2 | Forword | | AGGATAAGTACACGCTGCCC |
|  | Reverse | | TAACTGTCCATGCGCTGGTT |
| NANOG | Forword | | TGAGATGCCTCACACGGAGA |
|  | Reverse | | GCAGAAGTGGGTTGTTTGCC |
| REX1 | Forword | | ATGTGGACTACTCGGCCCTG |
|  | Reverse | | TGGACTCGTTGAAGATCCGC |
| OCT4 | Forword | | GCCCGAAAGAGAAAGCGAAC |
|  | Reverse | | AACCACACTCGGACCACATC |
| NESTIN | Forword | | TCAGGAGCAGCACTCTTAACTTACG |
|  | Reverse | | ATGAGATGGAGCAGGCAAGAGATTC |
| PAX6 | Forword | | GCAGAACAGTCACAGCGGAGT |
|  | Reverse | | TGATGGAGCCAGTCTCGTAATACCT |
| SOX1 | Forword | | CGGAGGAAGCGGAAAGCGTTT |
|  | Reverse | | GTGCTTGGACCTGCCTTACTACATT |
| TBX1 | Forword | | CGCAGTGGATGAAGCAAATCGTG |
|  | Reverse | | TTTGCGTGGGTCCACATAGACC |
| MSX1 | Forword | | CGAGAGGACCCCGTGGATGCAGAG |
|  | Reverse | | GGCGGCCATCTTCAGCTTCTCCAG |
| SOX17 | Forword | | ACGCTTTCATGGTGTGGGCTAAG |
|  | Reverse | | GTCAGCGCCTTCCACGACTTG |
| AFP | Forword | | GAATGCTGCAAACTGACCACGCTGGAAC |
|  | Reverse | | TGGCATTCAAGAGGGTTTTCAGTCTGGA |
| BRN3A | Forword | | GAGACAGAAGCAGAAGCGGATGAA |
|  | Reverse | | GCAGGCAGGATAACGGACACTC |
| VGLUT2 | Forword | | CAATCGAGCTGACGGAGGATGG |
|  | Reverse | | CGGTGGATGGTGCTGTTGTTGA |
| TRKB | Forword | | GCTGAATGCTATAACCTCTGTCCTG |
|  | Reverse | | AGACGCCATAGAACTTGACGATGT |
| NEUN | Forword | | TTCTATGCAGTGACGGGGTT |
|  | Reverse | | TCCATCCTGATACACGACCG |
| MAP2 | Forword | | CCCCTTGCTTCCCTGTAGAA |
|  | Reverse | | ATTTCCTCCTGGCAACCTCA |
| TUJ1 | Forword | | CTACAACGCCACGCTGTCCATC |
|  | Reverse | | CATCTGCTCGTCCACCTCCTTCA |
