# Supplementary figures and images for: Impaired AIF-CHCHD4 interaction and mitochondrial calcium overload contribute to auditory neuropathy spectrum disorder in patient-iPSC-derived neurons with AIFM1 variant
Source: Cell Death Dis. 2023 Jun 26;14(6):375. doi: 10.1038/s41419-023-05899-6 (PMC10293272; doi:10.1038/s41419-023-05899-6)

**Uncropped full-length gels and blot**


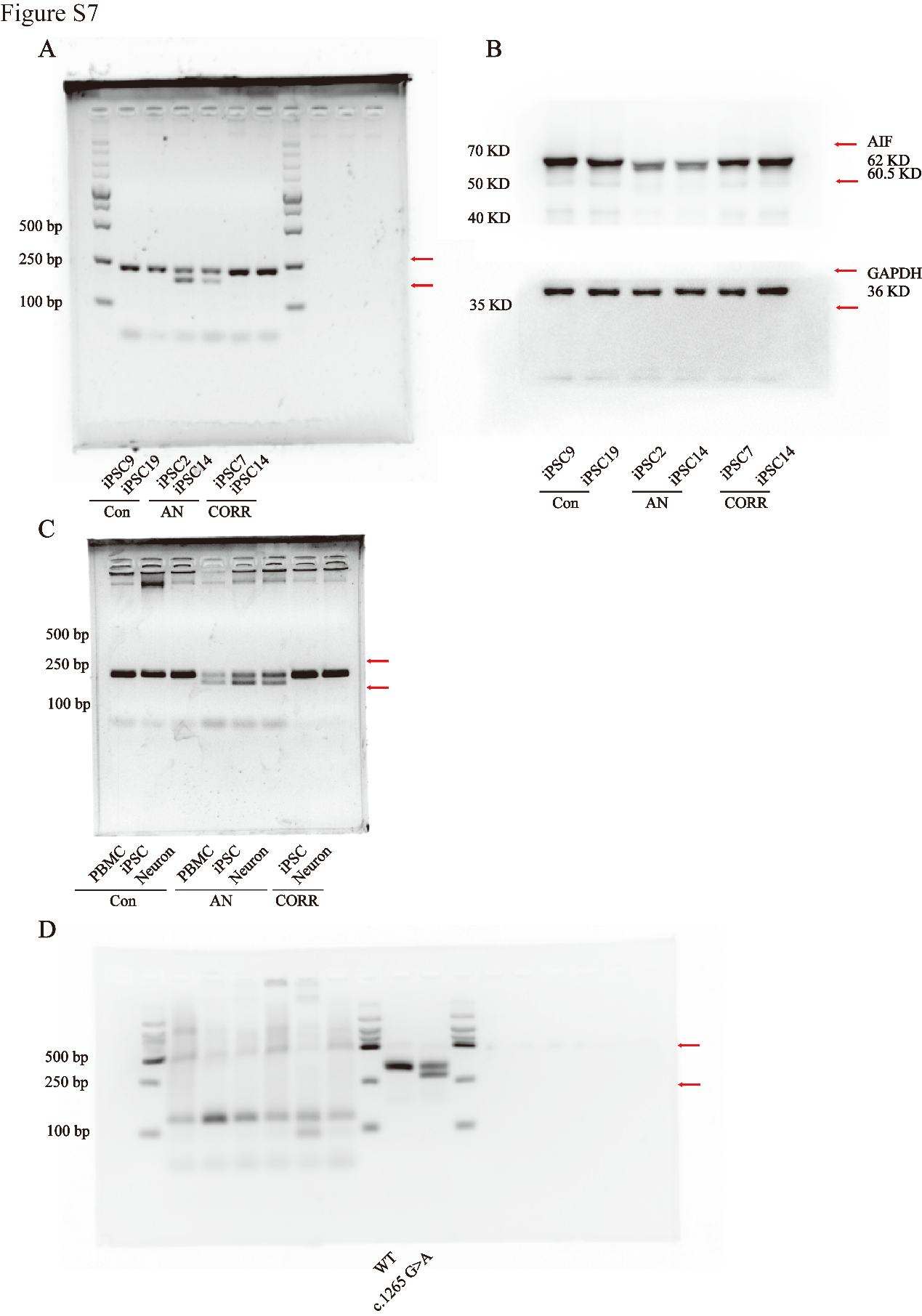


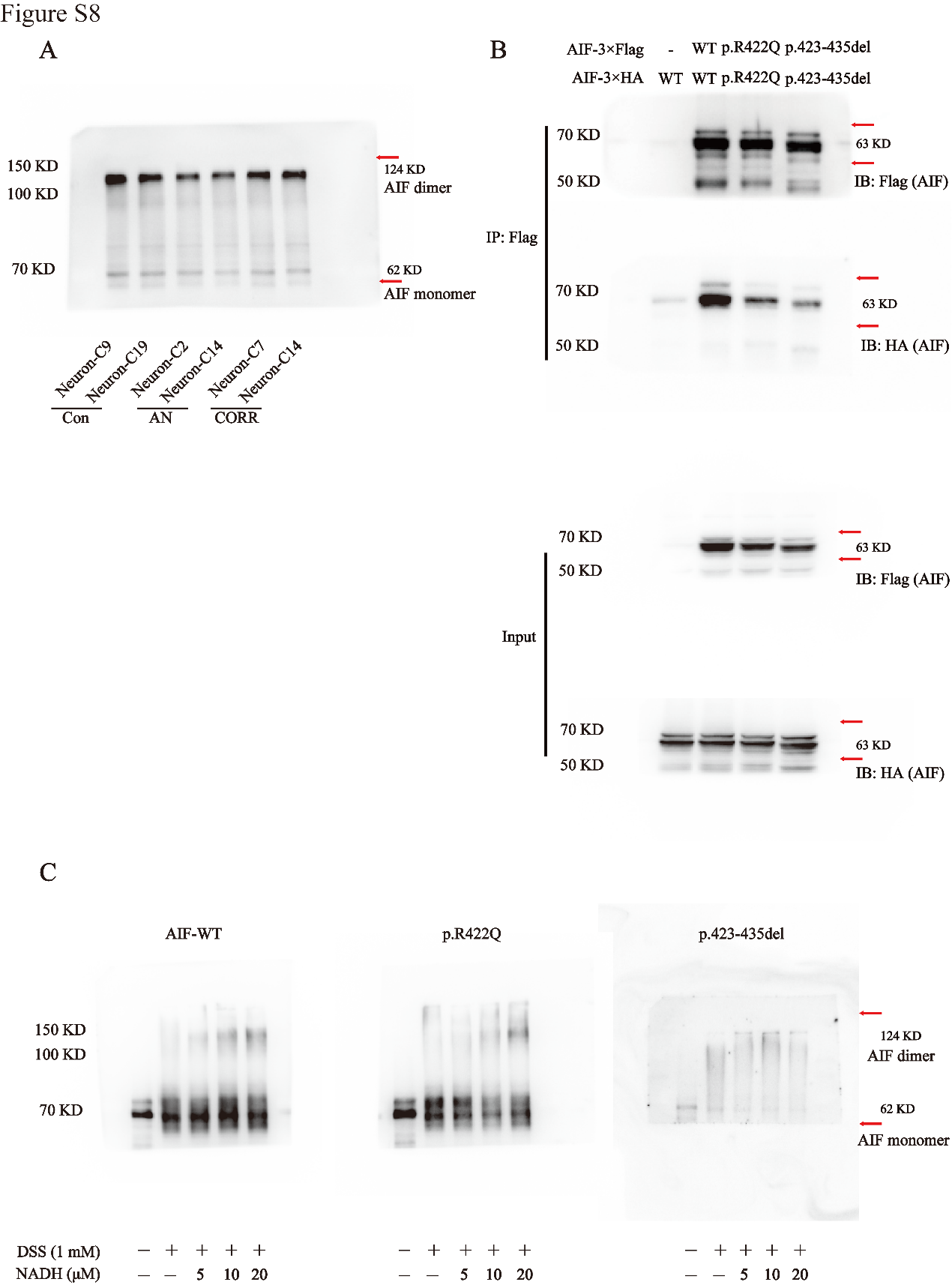


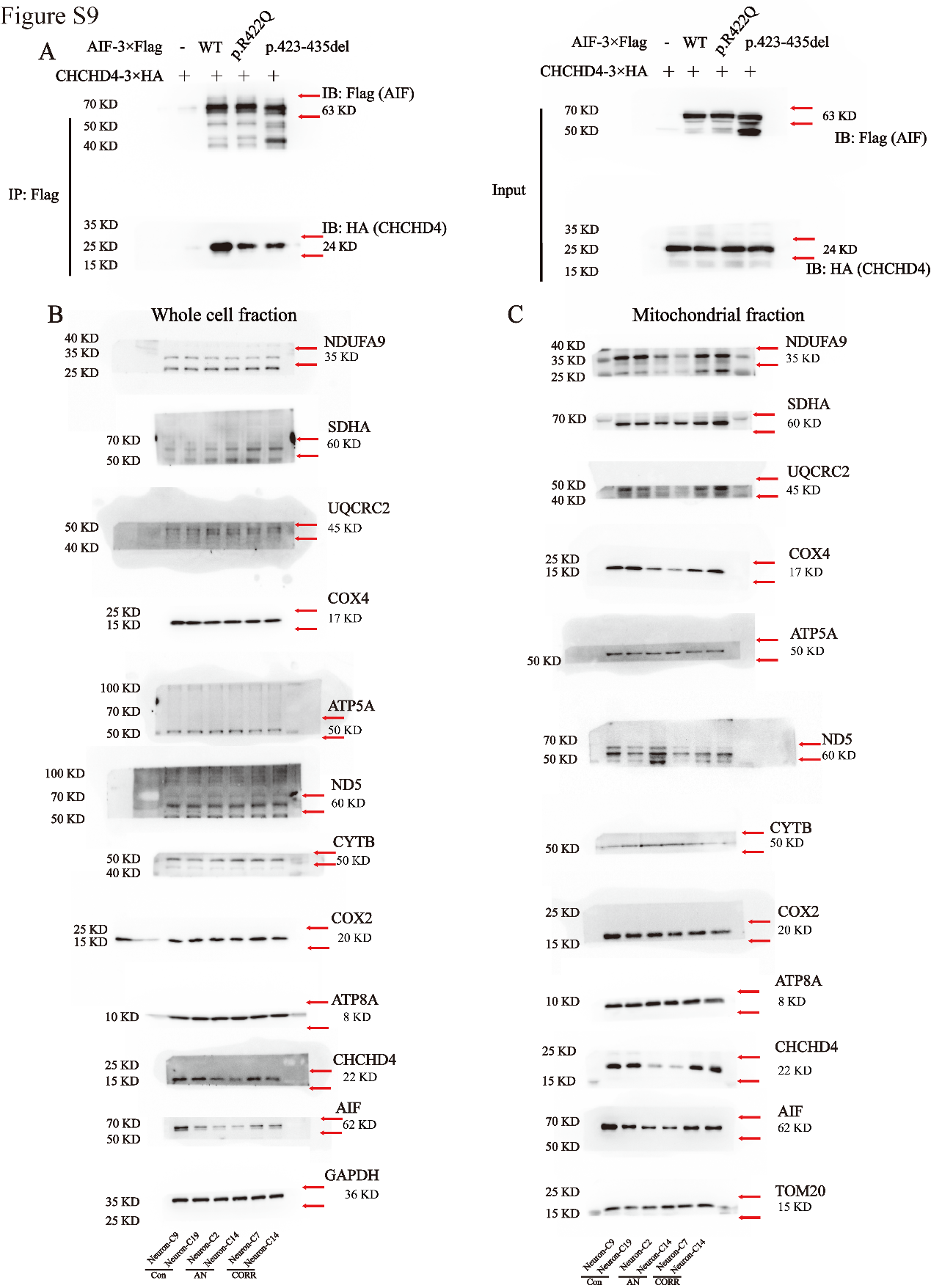


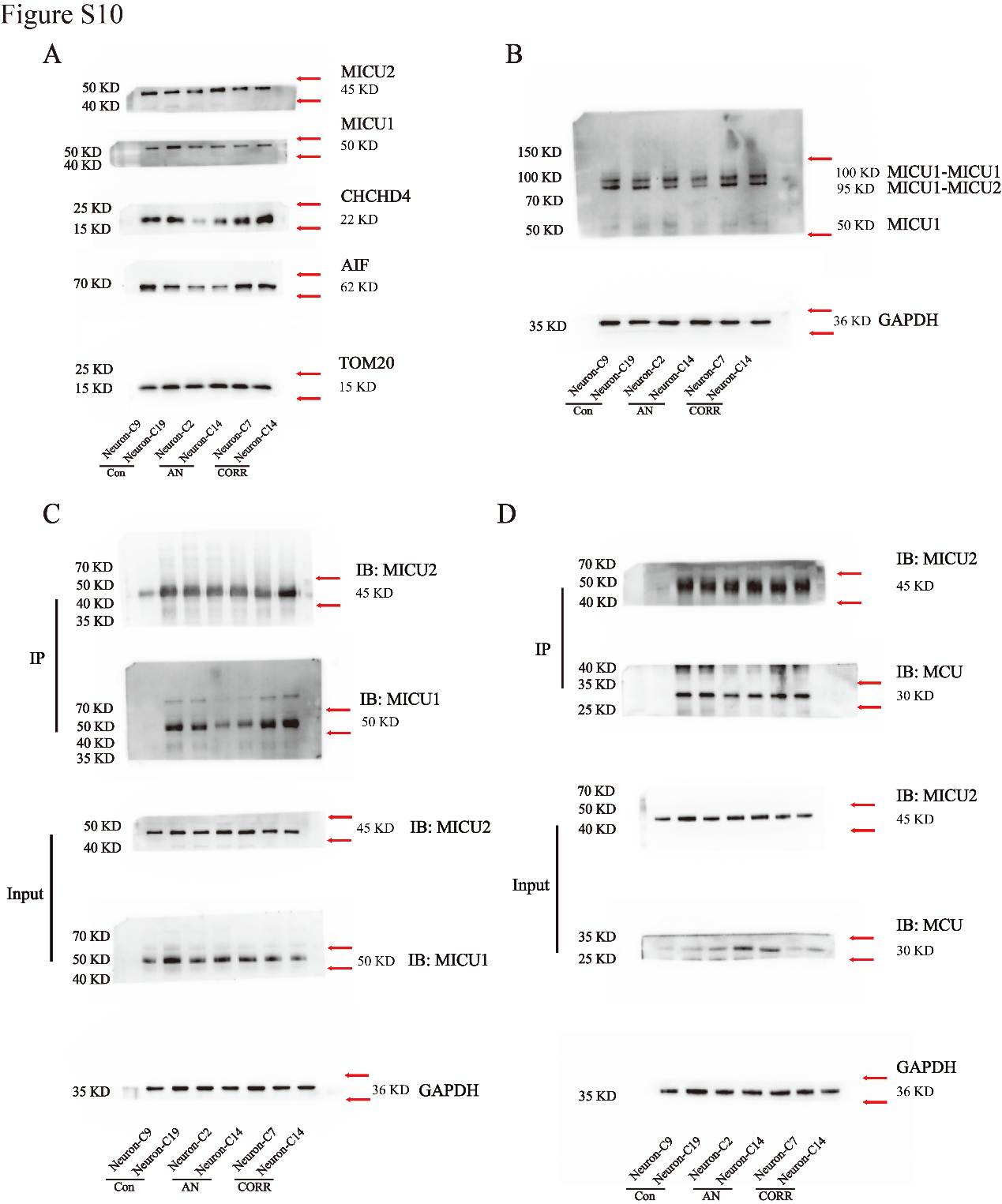


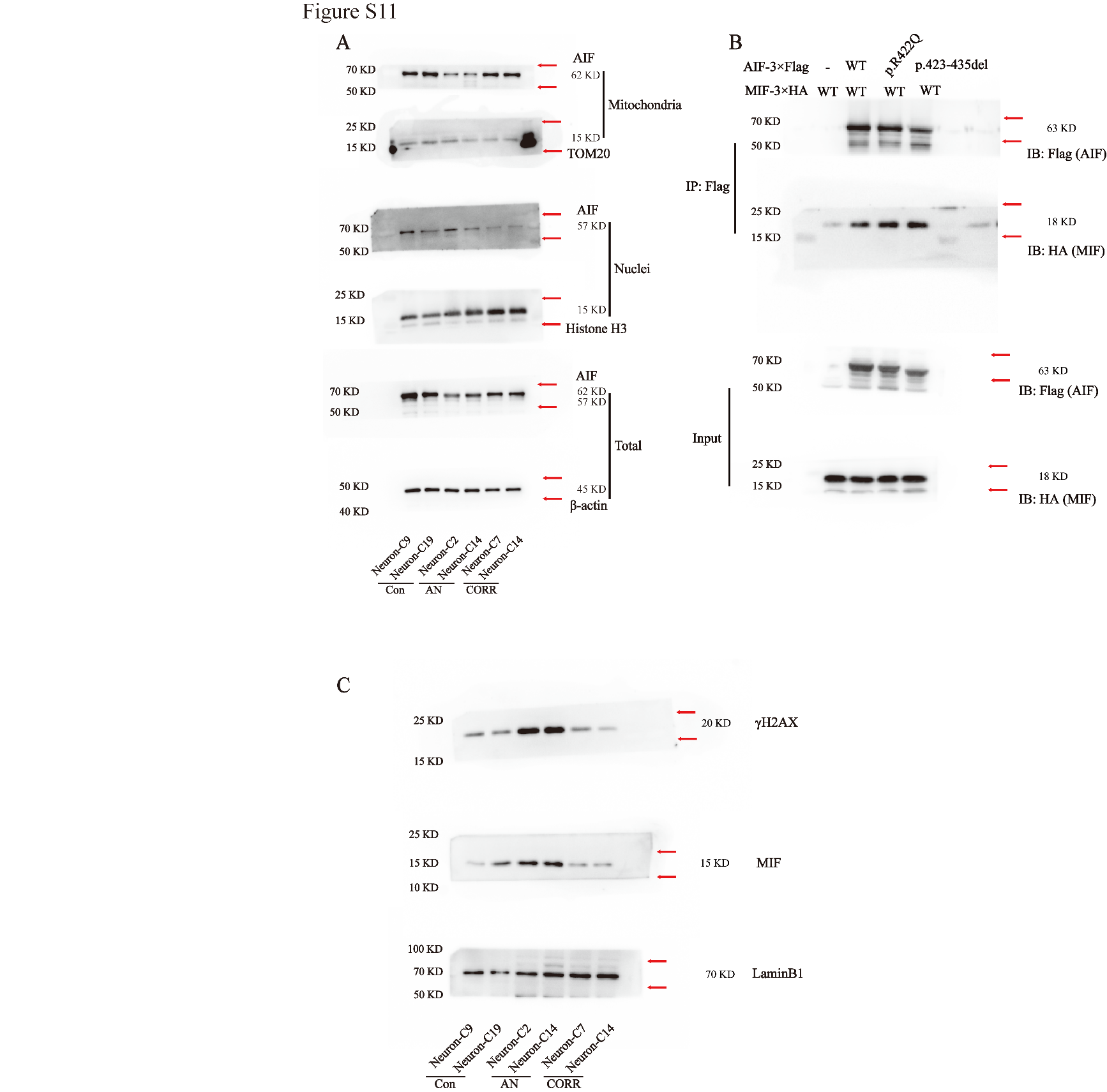


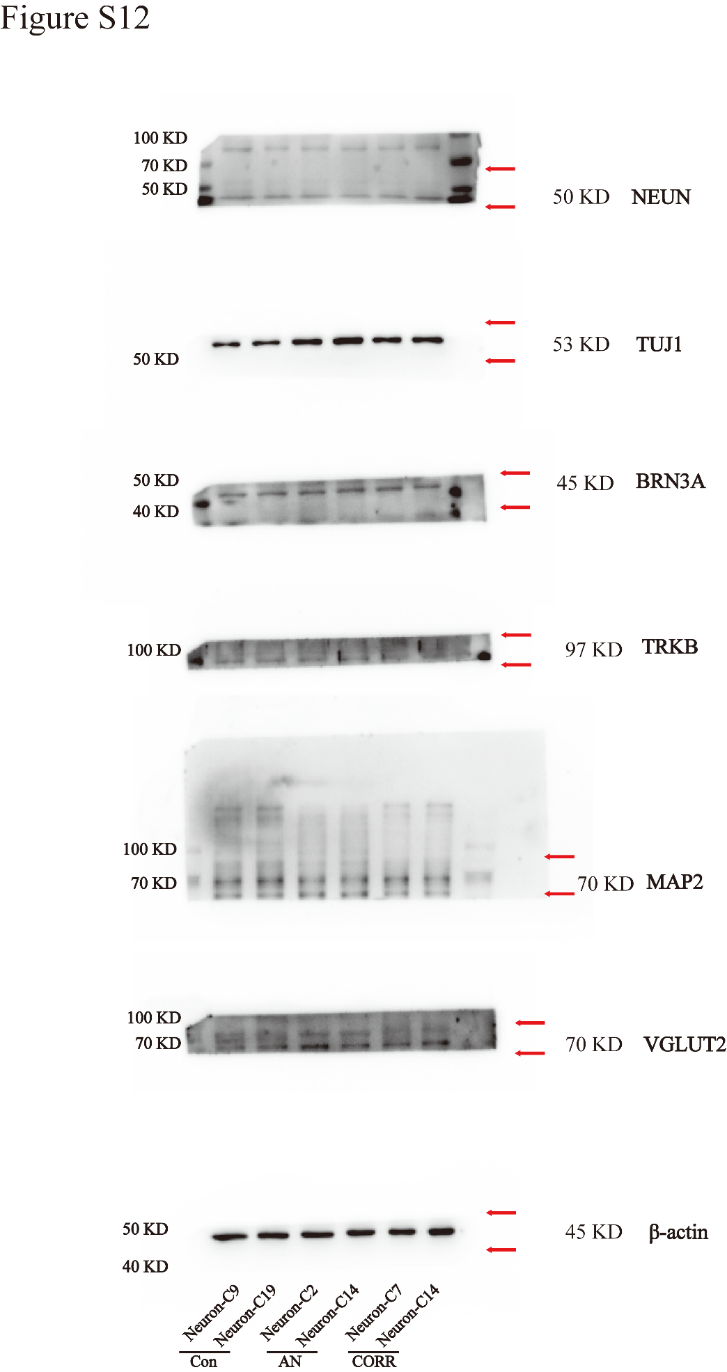


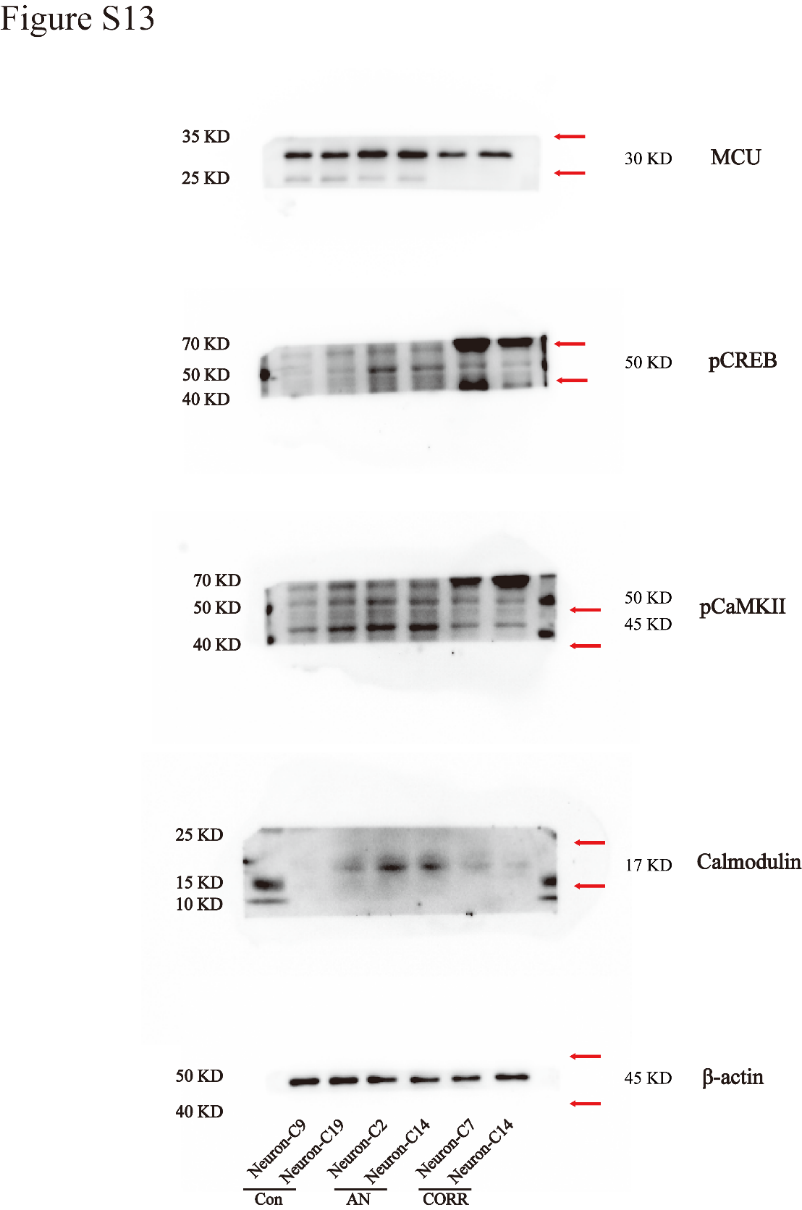


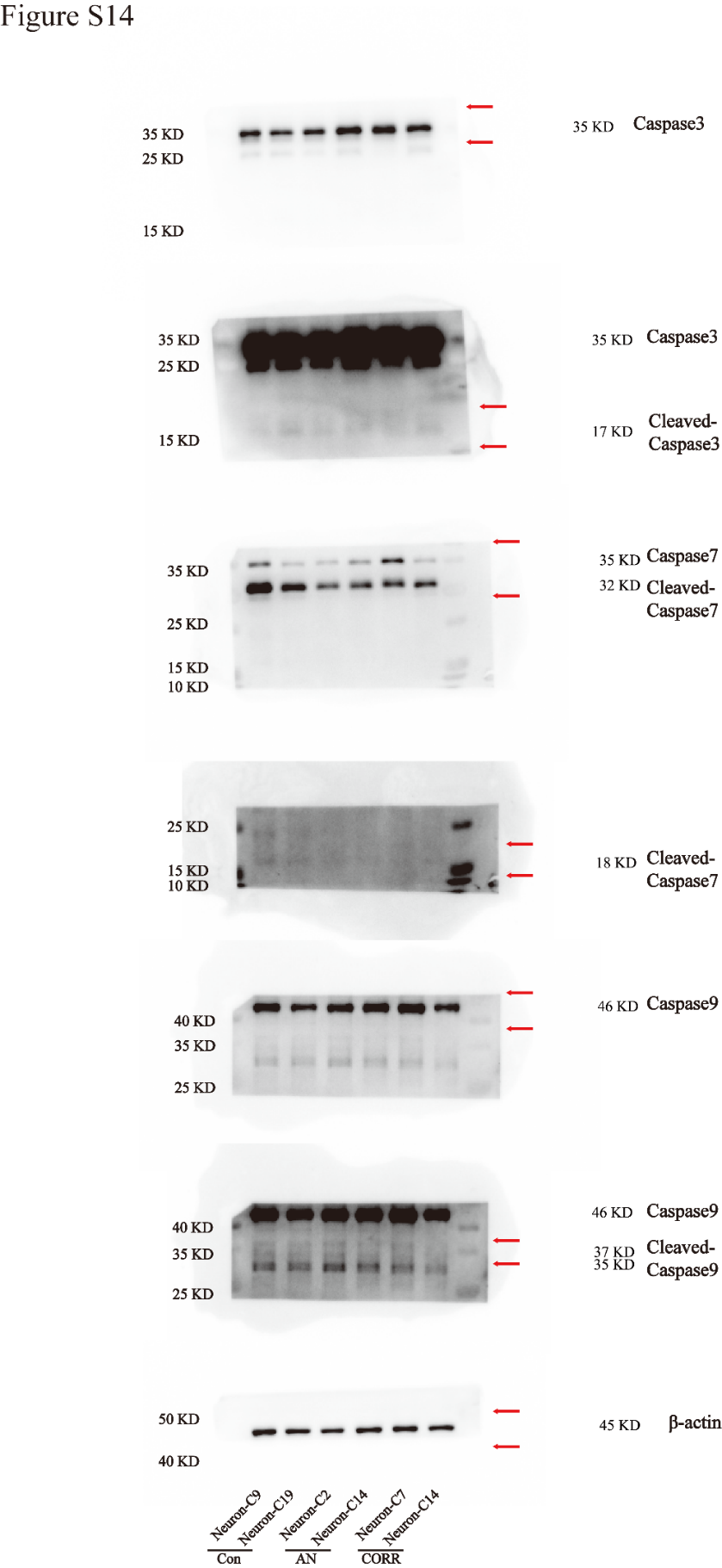

Supplement: Supplementary file 3 — Uncropped full-length gels and blot [file 41419_2023_5899_MOESM3_ESM.docx]
